# NOise Reduction with DIstribution Corrected (NORDIC) principal component analysis improves brain activity detection across rodent and human functional MRI contexts

**DOI:** 10.1162/imag_a_00325

**Published:** 2024-10-24

**Authors:** Russell W. Chan, Giles Hamilton-Fletcher, Bradley J. Edelman, Muneeb A. Faiq, Thajunnisa A. Sajitha, Steen Moeller, Kevin C. Chan

**Affiliations:** Department of Ophthalmology, New York University Grossman School of Medicine, New York, NY, United States; Neuroscience Institute, New York University Grossman School of Medicine, New York, NY, United States; Tech4Health Institute, New York University Grossman School of Medicine, New York, NY, United States; E-SENSE Innovation & Technology, Hong Kong, China; Hong Kong Centre for Cerebro-cardiovascular Health Engineering (COCHE), Hong Kong, China; Brain-Wide Circuits for Behavior Research Group, Max Planck Institute of Biological Intelligence, Planegg, Germany; Emotion Research Department, Max Planck Institute of Psychiatry, Munich, Germany; Center for Magnetic Resonance Research (CMRR), University of Minnesota, Minneapolis, MN, United States; Department of Radiology, New York University Grossman School of Medicine, New York, NY, United States; Department of Biomedical Engineering, Tandon School of Engineering, New York University, New York, NY, United States; Department of Ophthalmology, School of Medicine, University of Pittsburgh, Pittsburgh, PA, United States

**Keywords:** NORDIC, optogenetic fMRI, resting-state fMRI, population receptive field, cerebrovascular reactivity, thermal noise

## Abstract

NOise Reduction with DIstribution Corrected (NORDIC) principal component analysis (PCA) has been shown to selectively suppress thermal noise and improve the temporal signal-to-noise ratio (tSNR) in human functional magnetic resonance imaging (fMRI). However, the feasibility to improve data quality for rodent fMRI using NORDIC PCA remains uncertain. NORDIC PCA may also be particularly beneficial for improving topological brain mapping, as conventional mapping requires precise spatiotemporal signals from large datasets (ideally ~1 hour acquisition) for individual representations. In this study, we evaluated the effects of NORDIC PCA compared with “Standard” processing in various rodent fMRI contexts that range from task-evoked optogenetic fMRI to resting-state fMRI. We also evaluated the effects of NORDIC PCA on human resting-state and retinotopic mapping fMRI via population receptive field (pRF) modeling. In rodent optogenetic fMRI, apart from doubling the tSNR, NORDIC PCA resulted in a larger number of activated voxels and a significant decrease in the variance of evoked brain responses without altering brain morphology. In rodent resting-state fMRI, we found that NORDIC PCA induced a nearly threefold increase in tSNR and preserved task-free relative cerebrovascular reactivity (rCVR) across cortical depth. NORDIC PCA further improved the detection of TGN020-induced aquaporin-4 inhibition on rCVR compared with Standard processing without NORDIC PCA. NORDIC PCA also increased the tSNR for both human resting-state and pRF fMRI, and for the latter also increased activation cluster sizes while retaining retinotopic organization. This suggests that NORDIC PCA preserves the spatiotemporal precision of fMRI signals needed for pRF analysis, and effectively captures small activity changes with high sensitivity. Taken together, these results broadly demonstrate the value of NORDIC PCA for the enhanced detection of neural dynamics across various rodent and human fMRI contexts. This can in turn play an important role in improving fMRI image quality and sensitivity for translational and preclinical neuroimaging research.

## 1 Introduction

Functional magnetic resonance imaging (fMRI) is an indispensable tool for noninvasively mapping task-based brain activity (Bandettini et al., 1992; Kwong et al., 1992; Ogawa et al., 1992), resting-state functional connectivity (Van Essen & Ugurbil, 2012), and cerebrovascular reactivity (Chan et al., 2021; Liu et al., 2020) in humans. In preclinical settings, rodent fMRI is also valuable for translational and basic research, such as with using resting-state fMRI (rsfMRI) to identify changes in intrinsic brain-wide networks in disease models (van der Merwe et al., 2021). Rodent fMRI also provides comprehensive information to determine the global blood-oxygenation-level-dependent (BOLD) activation patterns in response to the manipulation of neural circuits using optogenetic fMRI (ofMRI) (Chan et al., 2017; Lee et al., 2010; Lin et al., 2016). Despite this utility, fMRI can suffer from a low signal-to-noise ratio (SNR), which presents a barrier for identifying statistically significant results. This is usually compounded by limitations in the quantity and quality of data acquisition, for example, when group-level analysis is composed of only a few animals, when experimental parameters affect the duration of individual scans, or when lower field strength MRI leads to noisier data. These limitations impede researchers’ ability to obtain high SNR data and may require substantial resources, such as additional animals and cost, to resolve.

One recent approach to improving the quality of rodent fMRI is community standardization, which is already common for clinical data acquisition, but has yet to be fully adopted in preclinical research. Despite differences in individual MR scanners, collaborative efforts can help identify and disseminate optimal acquisition parameters to many researchers across the field (Grandjean et al., 2023). An adjacent trend has also pushed for the standardization of fMRI data analysis pipelines for human (e.g. fMRIPrep) (Esteban et al., 2019), rodent (e.g. RABIES) (Desrosiers-Gregoire et al., 2022; Grandjean et al., 2020), and even nonhuman primate (e.g. Pypreclin) (Tasserie et al., 2020) research. These and other pipelines heavily focus on standardizing basic preprocessing principles such as slice-timing correction, head motion estimation, susceptibility distortion correction, and atlas/template registration, and often provide concise quality control reports. While these steps account for a large portion of the necessary analysis and are more or less similar across various rodent pipelines, there is a lack of consensus on other denoising and confound correction techniques. For example, techniques based on independent component analysis (e.g. ICA-AROMA, ICA-FIX) (Pruim et al., 2015; Zerbi et al., 2015) have been effectively utilized to correct vascular and/or motion-related noise. Additional nuisance regression approaches using signals from nonbrain regions have also proven effective at reducing additional sources of physiological noise (e.g. CompCor, GLMdenoise) (Behzadi et al., 2007; Chuang et al., 2019; Kay et al., 2013). However, although these algorithms have effectively increased the SNR of fMRI datasets, thermal noise is still largely overlooked in most pipelines.

Recently, NOise Reduction with DIstribution Corrected (NORDIC) principal component analysis (PCA) has demonstrated an ability to selectively suppress thermal noise and to significantly improve temporal SNR (tSNR) in human diffusion tensor imaging (Moeller et al., 2021) and fMRI (Vizioli et al., 2021). NORDIC PCA is constructed to specifically target Gaussian noise induced by the thermal sources in MRI system electronics, while leaving intact other non-Gaussian noise caused by neuronal fluctuations, respiration, etc. (Moeller et al., 2021; Vizioli et al., 2021). Other PCA approaches have been developed to separate these signal subspaces (Behzadi et al., 2007; Tierney et al., 2016); however, they often require a subjective definition of components that belong to each category and can, therefore, produce results that vary greatly across researchers and laboratories. This limitation was largely accounted for with the implementation of Marchenko-Pastur PCA (MPPCA) (Veraart et al., 2016). This approach provides an objective threshold of components based on the right edge of the Marchenko-Pastur distribution, which is a universal signature of noise in sample covariance matrices. Nevertheless, this approach has also been shown to lead to spatial blurring, which is undesirable in a field that continues to push for increasingly high spatial resolution data (Fernandes et al., 2023; Moeller et al., 2021). By contrast, NORDIC PCA also identifies an objective, data-driven threshold for the number of components to remove, but is specifically tuned to preserve spatial precision. As such, NORDIC PCA is a particularly promising thermal noise reduction technique that can complement other fMRI analysis pipelines. While NORDIC PCA has been tested and verified in a number of diverse human MRI contexts, the utility of this technique to improve various aspects of rodent fMRI analysis has yet to be extensively evaluated. Based on previous human fMRI results, we predict that the tSNR can be improved for rodent fMRI; however, it is currently unknown whether NORDIC PCA can also improve fMRI data quality for additional questions including but not limited to cerebrovascular reactivity and glymphatic system function.

In this study, we evaluated the effect of NORDIC PCA in an fMRI analysis pipeline for a range of applications that include rodent ofMRI and rsfMRI, as well as human receptive field mapping fMRI and task-free rsfMRI. We first evaluated the applicability of NORDIC PCA to ofMRI by quantifying the change in tSNR values, and by examining the overall effect on brain-wide BOLD activation maps. For rodent rsfMRI, we examined the effects of NORDIC PCA on resting-state BOLD signal characteristics such as tSNR and relative frequency content. As it has been suggested that tSNR and signal quality may be linked with relative cerebrovascular reactivity (rCVR) (Chu et al., 2018; Cohen et al., 2021) specifically by reducing signal variability (Vizioli et al., 2021), we also examined the effect of NORDIC PCA on task-free rCVR in different brain regions (Chan et al., 2021) and along cortical depth. Since neurovascular coupling can be altered by factors such as aquaporin-4 (AQP4) (Komaki et al., 2020; Nakada et al., 2017), we further investigated the effect of AQP4 inhibition on rCVR by measuring rsfMRI signals before and after intrathecal infusion of TGN020 (Vandebroek & Yasui, 2020), both with and without NORDIC PCA processing. Aside from rodent fMRI, we also applied NORDIC PCA to human population receptive field (pRF) mapping and rsfMRI data. Using human fMRI data can both verify the analysis pipeline utilized here for rodent fMRI and enable a within-study comparison with rodent results. This analysis also extends the evaluation of NORDIC PCA to additional human applications that may benefit from denoising techniques that preserve spatial precision. To address these aims, we evaluated the effect of NORDIC PCA on tSNR, retinotopic cluster size, and cortical organization, as well as on task-free rCVR in different brain regions. Overall, the results of this study can help improve fMRI image quality and sensitivity for translational and preclinical research.

## 2 Materials and Methods

### 2.1 Animal inclusion and preclinical study approval

Adult male ChAT-IRES-Cre knock-in mice (n = 4, 8–10 weeks old; The Jackson Laboratory: Strain #006410) and C57BL/6J mice (cortical depth analysis n = 13, 12–16 weeks old; AQP4 inhibition n = 3, 16 weeks old; The Jackson Laboratory: Strain #000664) were used for our ofMRI and rsfMRI experiments, respectively. Animals were housed under a 12-hour light/dark cycle with access to food and water *ad libitum*. All experimental procedures and animal husbandry were performed in strict accordance with the U.S. National Institutes of Health and New York University Grossman School of Medicine Institutional Animal Care and Use Committee guidelines.

### 2.2 Stereotaxic virus injection for ofMRI experiments

**Fig. 1.**
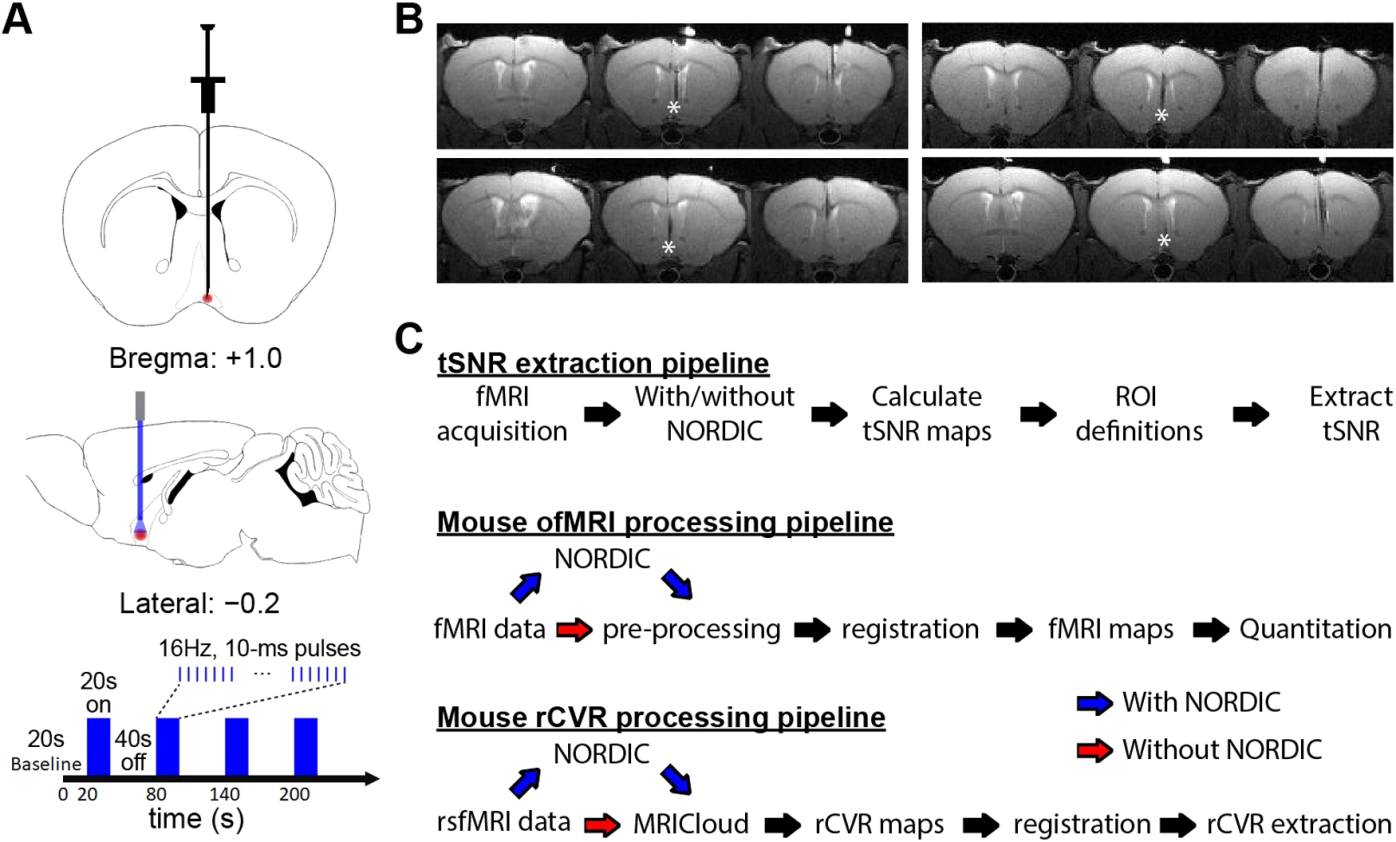
Viral injection, fiber implantation, and block-design stimulation for optogenetic functional fMRI (ofMRI) enable mapping of brain-wide cholinergic basal forebrain networks, and processing pipelines to evaluate the effects of NORDIC-correction in mouse ofMRI and resting-state functional MRI (rsfMRI)-derived rCVR. (A) 1.0 μL of the AAV5/DIO-ChR2–EYFP virus was injected to the basal forebrain (+ 1.0 mm AP, − 0.2 mm ML, + 5.2 mm DV) at a 50 nL/min flow rate, after which a custom-designed fiber optic cannula was implanted. After at least 4 weeks, optogenetic fMRI experiments were performed using a block-design stimulus consisting of blue light (473 nm) at 16 Hz. Each run consisted of a 20 sec baseline, followed by 4 blocks with 20 sec of stimulation and 40 sec of subsequent rest. (B) Cannula locations were validated in all animals used for the optogenetic fMRI experiments with T2-weighted structural MRI. * indicates the location of the fiber tip. (C) Processing pipelines used to evaluate the effects of NORDIC PCA on tSNR (top), ofMRI activation (middle), and rsfMRI-derived rCVR (bottom).

For ofMRI experiments (Chan, Cron, et al., 2022; Chan, Lee, et al., 2022; Chan et al., 2017; Lee et al., 2010), a double-floxed inverted open reading frame (DIO) recombinant adeno-associated virus-5 (AAV5) was used to express ChR2-EYFP in Cre-expressing neurons. The recombinant AAV vector was packaged by the University of North Carolina viral vector core at a titer of 4 × 10^12^ particles/mL. Animals were anesthetized with isoflurane (induction 3%, maintenance 1.5%–2%) and secured in a stereotactic frame with nonrupturing ear bars. A heating pad was used to maintain body temperature at 37°C, and sterile ocular lubricant was applied to the eyes to prevent desiccation during surgery. Buprenorphine (0.1 mg/kg) was injected subcutaneously for analgesia. After a midline incision in the scalp, a small craniotomy was created using a dental drill, followed by virus injection and cannula implantation in the basal forebrain (+ 1.0 mm AP, − 0.2 mm ML, and + 5.2 mm DV, according to the Allen Brain Atlas: https://www.brain-map.org; Fig. 1A). Then, 1.0 μL of the AAV5/DIO-ChR2–EYFP virus was injected at 50 nL/min, driven by a microsyringe pump controller. After injection, the needle was held in place for 10 min before slowly being retracted from the brain. A custom-designed 200 μm diameter fiber optic cannula was mounted and secured on the skull using light-cured dental cement (Kuraray Inc.), with the optical fiber extending from the cannula’s base to the desired depth at 0.1 mm above the injection site (Fig. 1A). Following surgery, mice were kept on a heating pad until recovery from anesthesia and were given carprofen (5 mg/kg, subcutaneously) daily for 2 days as postoperative analgesia to minimize discomfort. All fMRI experiments were conducted at least 4 weeks following virus injection to ensure proper ChR2 expression. Optical fiber location was validated in all animals used for ofMRI experiments by examining T2-weighted structural MRI images (Fig. 1B).

### 2.3 Intrathecal surgery for rsfMRI of aquaporin-4 inhibition

For AQP4 inhibition rsfMRI experiments, a polyethylene tubing (inner diameter = 0.28 mm; outer diameter = 0.60 mm) was surgically placed intrathecally at the lumbar region (L4–L5) of healthy adult mice. TGN020 (30 mg/kg) was infused at a rate of 1.6 μL/min for 30 min.

### 2.4 MRI acquisition—rodent ofMRI and rsfMRI

All rodent MRI experiments were carried out on a 7-Tesla Bruker Biospec (70/30) small animal MRI system at the Preclinical Imaging Laboratory core facility at New York University Grossman School of Medicine using a transmit-only birdcage coil in combination with (1) a custom-designed actively decoupled 1 cm diameter receive-only surface coil for ofMRI experiments or (2) a Bruker 1 H four-channel phased array receive-only CryoProbe for rsfMRI experiments (half-shell inner diameter = 20 mm; length = 85 mm). Animals were initially anesthetized in an induction chamber with 3% isoflurane and were provided an initial subcutaneous (s.c.) bolus of 0.1 mg/kg dexmedetomidine before being placed onto the MRI-compatible cradle with ears, teeth, and head secured. To maximize SNR, the receiver coil was positioned on top of the head and centered over the brain, after which the animal was placed into the isocenter of the magnet. Throughout scanning, animals were lightly anesthetized and sedated using a combination of low-dose isoflurane (0.25% isoflurane) and dexmedetomidine (continuous s.c. infusion at 0.1 mg/kg/hour). During all experiments, animal temperature was maintained throughout fMRI acquisition using a heated circulating water bath. Continuous physiological monitoring was also performed using an MRI-compatible system (SA Instruments). Vital signs were within normal physiological ranges (rectal temperature: 36.5–37.5°C, heart rate: 260–420 beat/min, breathing rate: 80–120 breath/min, and oxygen saturation: >90%) throughout the duration of the experiments. Heart rate was stable for individuals and only varied across animals, but in general was within the same range that has been reported elsewhere (You et al., 2021).

For ofMRI, 20 contiguous 0.75 mm thick coronal slices were positioned to cover the whole brain. T2-weighted high-resolution anatomical images were acquired prior to ofMRI to check for brain damage and to confirm accurate fiber location. These anatomical images were acquired using a rapid acquisition with relaxation enhancement (RARE) sequence with RARE factor = 4, echo time (TE)/repetition time (TR) = 8.3/3000 ms, field of view (FOV) = 16 x 16 mm^2^, and 160 x 160 matrix size. ofMRI measurements were obtained for the same slice locations using a single-shot gradient-echo echo-planar-imaging (GE-EPI) sequence with TE/TR = 12/1000 ms, FOV = 16 x 16 mm^2^, 64 x 64 matrix size, voxel size (VOX) = 0.25 x 0.25 x 0.75 mm^3^, receiver bandwidth = 3125 Hz, and number of repetitions = 260. To reduce acquisition time, we utilized partial Fourier k-space-encoding acceleration in the phase-encoding dimension using an acceleration factor = 1.2, overscans = 12, and in the frequency-encoding dimension using an acceleration factor = 1.0 and overscans = 40.

For rsfMRI, 30 contiguous 0.5 mm thick coronal slices were positioned to cover the whole brain. T2-weighted high-resolution anatomical images were acquired prior to rsfMRI as an anatomical reference. These anatomical images were acquired using a RARE sequence with RARE factor = 4, TE/TR = 8.3/3750 ms, FOV = 16×16 mm^2^, and 160 × 160 matrix size. rsfMRI measurements were obtained for the same slice localizations using a single-shot GE-EPI sequence with TE/ TR = 12/1000 ms, FOV = 16 x 7 mm^2^, 80 x 35 matrix size, VOX = 0.20 x 0.20 x 0.50 mm^3^, receiver bandwidth = 2500 Hz, and number of repetitions = 600. These rsfMRI data were acquired with the same partial Fourier k-space-encoding acceleration as was performed for the ofMRI dataset.

For ofMRI experiments, the MR scanner and laser for optogenetic stimulation were synchronized using an Arduino Uno with custom codes. The light delivery system was maintained outside of the scanner room and long optical cables (<5 m) were used to deliver light into the implanted fibers of the mouse in the scanner bore. Blue light was delivered using a 473 nm DPSS laser that was calibrated prior to scanning to deliver ~1.5 mW at the fiber tip (200 μm). Optogenetic stimulation was delivered at 16 Hz with a pulse width of 10 ms (i.e., 16% duty cycle), as these parameters have previously been shown to robustly activate the cholinergic basal forebrain circuit (Xu et al., 2015). A block-design paradigm was employed that began with a 20 sec baseline and was then followed by four 20-sec stimulation periods that repeated every 60 sec (40 sec nonlight period) (Fig. 1A).

For AQP4 inhibition rsfMRI experiments, animals were scanned immediately before and 30 min after intrathecal infusion of the AQP4 inhibitor TGN020.

### 2.5 Data analysis—rodent ofMRI and rsfMRI

Standard preprocessing using SPM12 (www.fil.ion.ucl.ac.uk/spm) was applied to raw ofMRI and rsfMRI magnitude images with NORDIC PCA or without (Standard) (github.com/SteenMoeller/NORDIC_Raw). In both cases, the first 10 brain volumes (dummy volumes) were removed from each dataset prior to additional processing steps to allow the magnetic field to reach equilibrium. The NORDIC PCA algorithm for magnitude images follows the same concept as for complex images, that is, noise flattening followed by spatially invariant noise-based thresholding using an 11:1 ratio of spatial to temporal voxels. Previous work using numerical simulations, diffusion MRI and fMRI, has found this ratio to be optimal (Moeller et al., 2021; Vizioli et al., 2021). This ratio led to a kernel size of [14 14 14] and [19 19 19] for the ofMRI and rsfMRI datasets, respectively. In a separate analysis, we also directly compared NORDIC and MPPCA using the same kernel size. For this we used a kernel size of [7 7 7] for both methods as this is the established parameter space for MPPCA. The spatial noise in the images was flattened using a spatial noise estimate with the Marchenko-Pastur distribution asymptotic properties. The threshold in NORDIC PCA patch-based denoising for magnitude images was set proportional to the largest singular value from a Gaussian distribution for a patch of equal size. Patch overlapping and averaging with 1/2 the patch size shift between patches were used. After NORDIC PCA, fMRI image timestamps were adjusted to account for slice timing differences. This was necessary since for a given time point (i.e. a single repetition or TR), slices were acquired one after another rather than simultaneously. Brain extraction was performed using the Amira software (Thermo Fisher Scientific). Standard and NORDIC-processed data were then motion corrected using a 6-parameter rigid registration procedure and smoothed using a Gaussian kernel with full width at half maximum of 2 voxels to remove local sources of white noise. Data were linearly detrended to minimize baseline drift caused by system instability. Briefly, a global linear trend was first calculated using linear regression of the temporal signal obtained from the whole brain. Then, this global linear trend was subtracted from the temporal signal of each voxel. fMRI data were then temporally band-pass filtered (ofMRI: 0.001–0.25 Hz, rsfMRI: 0.001–0.1 Hz) to reduce physiological noise. T2-weighted images from each animal were registered to a custom-made brain template acquired with the same settings. As inbred mouse lines such as those used in the current work exhibit a high level of anatomical consistency (Badea et al., 2007; Kovačević et al., 2005), registration was performed using an affine transformation (Chan, Cron, et al., 2022; Chan, Lee, et al., 2022; Chan et al., 2017; Lee et al., 2010). The quality of the registration was performed manually to ensure that there were no major errors in alignment.

Whole-brain tSNR maps were calculated by dividing voxel-wise mean by voxel-wise standard deviation of the BOLD timeseries. In addition, focal tSNR values were extracted from anatomical regions of interest (ROIs) defined across cortical and subcortical areas according to the Allen Brain Atlas. These regions include the somatosensory cortex (SS), cingulate cortex (Cg), superior colliculus (SC), lateral geniculate nucleus (LGN), and basal forebrain (BF).

For ofMRI, a double gamma basis set was convolved with the stimulation block design and a fixed-effects general linear model (GLM) was used to produce activation maps. To account for the sharper hemodynamic response characterized in rodents (Lambers et al., 2020; Pisauro et al., 2013; You et al., 2021), we utilized a hemodynamic response function (HRF) with the following values: delay of response = 3.7 sec, delay of undershoot = 4.45 sec, dispersion of response = 0.5 sec, dispersion of undershoot = 0.5 sec, ratio of response undershoot = 1.5 sec, onset = 0 sec, and kernel length = 32 sec. Quality control was performed by manually assessing the GLM results from each animal before being included in the group-level analysis. BOLD timeseries were extracted from predefined anatomical areas by averaging the timeseries of all voxels within the ROI. The standard deviation of the BOLD timeseries was also extracted from each ROI. For rsfMRI, the power spectrum of the BOLD timeseries was extracted from each predefined ROI.

MRICloud (mricloud.org/) was used to generate the whole-brain rCVR maps from the rsfMRI data (Chan et al., 2021; Chan, Lee, et al., 2022; Liu et al., 2017, 2022; Lu et al., 2014). Specifically, the CVR-MRICloud tool accepts raw ANALYZE files as an input and applies a default processing pipeline that includes motion correction, spatial smoothing, linear detrending, and temporal filtering (Liu et al., 2017, 2022). As such, we used CVR-MRICloud on raw data with and without the application of NORDIC PCA (Fig. 1C). The voxel-wise rCVR index (*α*) was first computed using a GLM containing the normalized BOLD timeseries (*ΔBOLD/BOLD*) and the global signal timeseries (*GS*). *ΔBOLD/BOLD* was computed according to the following equation:

$$\frac{\Delta BOLD}{BOLD}=\left(s-mean\left(s\right)\right)/\left(\frac{2\left|s-mean\left(s\right)\right|}{\sqrt{N)}}\right),$$

where *s* is the detrended and filtered BOLD timeseries and *N* is the total number of dynamics. This rescaling ensures that the timeseries has a zero mean and norm of $\sqrt{N}/2$ (Liu et al., 2022). Voxel-wise rCVR values were then obtained by normalizing *α* by the tissue signal intensity averaged across the whole brain (*SI*). These steps can be summarized as follows (note the residual term *β* was not used in this analysis):

$$\begin{array}{l} rCVR=\frac{\alpha }{SI},where\;\alpha \;is\;obtained\;from\;\frac{\Delta BOLD}{BOLD}\\ \;\;\;\;\;\;\;\;\;\;\;\;\;=\alpha \cdot GS+\beta . \end{array}$$

Anatomically constrained rCVR values were extracted using the same previously described ROIs as well as along the cortical depth, as defined by the Allen Brain Atlas.

### 2.6 Clinical study approval and human subject recruitment

Healthy human subjects were recruited for both pRF fMRI (n = 7, 48–70 years old) and rsfMRI (n = 21, 41–79 years old). The institutional review board and ethics committee of the University of Pittsburgh approved this study and all subjects provided written informed consent. This study followed the tenets of the Declaration of Helsinki and was conducted in compliance with the Health Insurance Portability and Accountability Act.

### 2.7 MRI acquisition—human pRF fMRI and rsfMRI

Human MRI experiments were performed on a 3-Tesla Allegra head scanner (Siemens, Erlangen, Germany) at the Neuroscience Imaging Center at the University of Pittsburgh. Initially, a conventional whole-brain T1-weighted anatomical MRI was acquired using a 3D MPRAGE pulse sequence with 176 contiguous 1.0 mm thick sagittal slices, TE/TR = 2.5/1400 ms, inversion time (TI) = 800 ms, flip angle = 8°, FOV = 25.6 x 25.6 x 17.6 cm^3^, 256 x 256 matrix size, and VOX = 1.0 x 1.0 x 1.0 mm^3^.

**Fig. 2.**
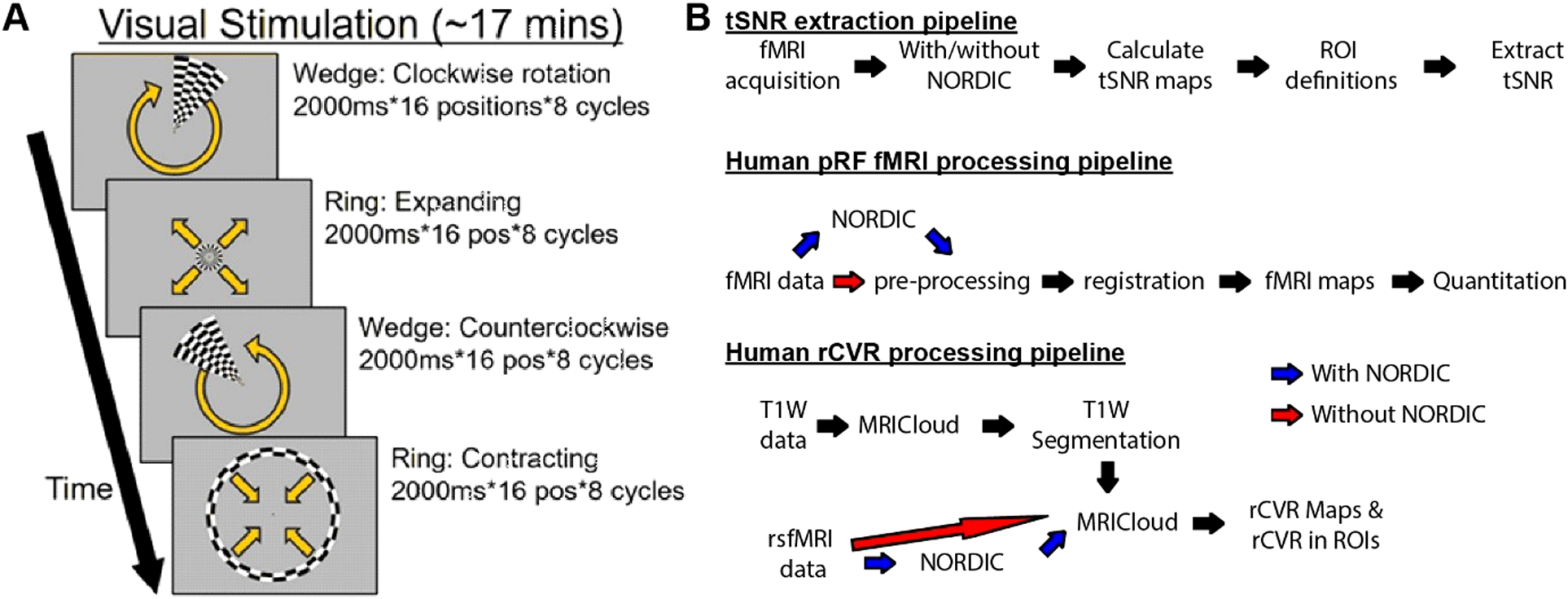
Visual stimulation during task-based fMRI experiments for retinotopic mapping via population receptive field modeling, and processing pipelines used to evaluate the effects of NORDIC PCA in human fMRI. (A) Subjects positioned inside the MRI scanner observed ~17 min of visual stimulation, consisting of four conditions that involved either a moving checkerboard ring moving clockwise/counter-clockwise or a wedge expanded/contracted, respectively (yellow arrows). These four conditions consisted of single image presentations lasting 2000 ms, sequentially moving across 16 positions and repeating 8 cycles per condition (1024 sec total). Visual stimulation was presented on a screen inside the scanner that subtended a 26° visual angle. Subjects were tasked with responding using a key press on a provided keypad when a central fixation dot changed color. (B) Processing pipelines used to evaluate the effects of NORDIC PCA on tSNR (top), pRF fMRI (middle), and rsfMRI-derived rCVR (bottom).

For task-based pRF fMRI, T2*-weighted whole-brain BOLD images were collected using a single-shot GE-EPI pulse sequence with 28 contiguous 3.24 mm thick axial slices, TE/TR = 26/2000 ms, FOV = 20.5 x 20.5 cm^2^ (positioned to ensure coverage of the bilateral visual cortices), 104 x 104 matrix size, VOX = 2.0 x 2.0 mm^2^, and number of repetitions = 512. During T2*-weighted fMRI scans, subjects underwent retinotopic stimulation via visual presentation of checkerboard rings and wedges on a screen with a 26° field of view. Subjects were asked to fixate on the center of the image and respond with a key press when a small central dot changed. During this procedure, subjects were presented with four different conditions: (1) wedge rotating clockwise, (2) ring expanding, (3) wedge rotating counter-clockwise, and (4) ring contracting. Each of these conditions involved displaying an image for 2 sec, with 16 positions per repetition and 8 repetitions per scan (total scan length = 17 min and 4 sec) (Fig. 2A).

rsfMRI was performed while the participant remained at rest with his/her eyes closed. T2*-weighted BOLD images were collected using a single-shot GE-EPI pulse sequence covering the whole brain with 38 contiguous 3 mm thick axial slices, TE/TR = 26/2000 ms, FOV = 20.5 x 20.5 cm^2^, 64 x 64 matrix size, VOX = 2.0 x 2.0 x 3.0 mm^2^, and number of repetitions = 240.

### 2.8 Data analysis—human pRF fMRI and rsfMRI

Similar to the rodent fMRI data analysis, whole-brain tSNR maps were calculated from the human pRF fMRI and rsfMRI data with and without the application of NORDIC PCA by dividing the voxel-wise mean by the voxel-wise standard deviation of the BOLD timeseries (Fig. 2B). NORDIC PCA was only applied to magnitude images with the same default settings as was used for the rodent fMRI datasets (github.com/SteenMoeller/NORDIC_Raw). In using the default 11:1 ratio of spatial to temporal voxels, the kernel size for the pRF fMRI and rsfMRI datasets was [18 18 18] and [14 14 14], respectively. As with the rodent fMRI analysis, we also compared NORDIC and MPPCA in a separate analysis using a kernel size of [7 7 7] for both methods.

For pRF maps, fMRI volumes were initially processed either without (“Standard”) or with NORDIC PCA. The Standard and NORDIC-processed images were then preprocessed by applying brain extraction, slice-timing correction, and 3D motion correction. Temporal filtering was not applied to pRF data as this processing step has previously been shown to artificially increase pRF estimates and bias the interpretation of spatial tuning properties (Morgan & Schwarzkopf, 2020). This was followed by coregistration with the corresponding T1-weighted anatomical image, and normalization into the Montreal Neurological Institute (MNI) space using BrainVoyager’s default processing pipeline. The pRF analysis was performed according to the method described by Dumoulin and Wandell (2008) and implemented in the BrainVoyager software using default parameter settings. Briefly, the pRF mapping procedure modeled the expected hemodynamic response profile for receptive fields at different locations and sizes in the visual field during the presented visual stimulation. Individual GLMs were computed for every combination of the following parameters: 30 horizontal locations, 30 vertical locations, and 30 sizes (0.20–7.00°). Each of these GLMs produced a corresponding value for the explained variance (R^2^), and a winner-take-all approach was applied such that the model with the highest R^2^ was assigned to that voxel. The receptive field location (X, Y) and size that corresponded to the best fitting GLM were then linked to each voxel, which was also used to compute the associated eccentricity and polar angle values. For visualization purposes, pRF maps were displayed in cortical surface space and were thresholded at R > 0.2 (rather than R^2^). Cluster sizes were reported after cluster extent thresholding to correct for multiple comparisons (see Section 2.9).

Human rsfMRI data were analyzed using MRICloud (mricloud.org/) to generate whole-brain rCVR maps using the same default processing pipeline previously described for rodent rsfMRI data (see Section 2.5 for details) (Chan, Lee, et al., 2022; Chan et al., 2021; Liu et al., 2017, 2022; Lu et al., 2014). Whole-brain rCVR maps in the registered MNI space were averaged between subjects for display. T1-weighted images were also segmented using *T1 MultiAtlas Segmentation* in MRICloud with default settings to extract various anatomical ROIs (Fig. 2B). Group-averaged rCVR values were extracted from the ROIs covering the visual cortex (VC), motor cortex (MC), caudate putamen (CPu), hippocampus (HP), and basal forebrain (BF).

### 2.9 Statistical analysis

Statistical analysis was performed using a combination of MATLAB, SPSS, and GraphPad PRISM. In general, results are presented as mean ± standard error of the mean (S.E.M.) across subjects. Unpaired and paired student’s *t*-tests were applied with a false discovery rate or Bonferroni correction for multiple comparisons. For scatter plots, simple linear regression was employed to test significance. Results are considered significant when *p* < 0.05. We denote *p < 0.05, **p < 0.01, and ***p < 0.005. For mouse ofMRI maps, a Student’s *t*-test was performed to identify activated voxels using the threshold *p* < 0.001, false discovery rate corrected for multiple comparisons. Human pRF maps are displayed at a threshold of R > 0.2 (square root of variance explained of the best fitting model), corrected for multiple comparisons using cluster extent thresholding (Woo et al., 2014). The minimum cluster size was derived empirically using Monte Carlo simulations (1000 iterations) with α = 0.05. All values described here are default parameter values provided in the BrainVoyager software and the ClusterThresh plugin.

## 3 Results and Discussion

### 3.1 NORDIC PCA for rodent ofMRI and rsfMRI

**Fig. 3.**
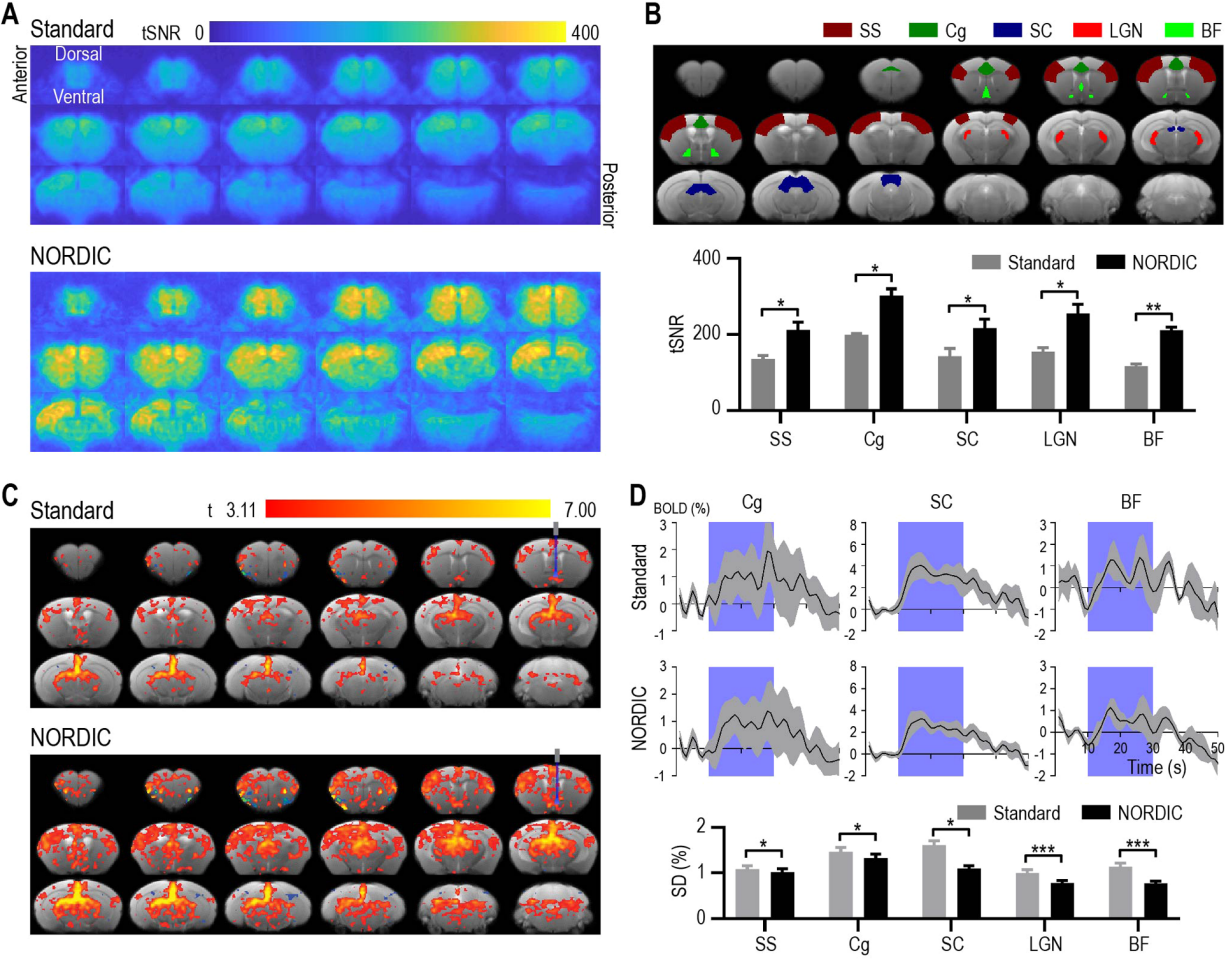
NORDIC PCA increased the temporal signal-to-noise ratio (tSNR) and number of activated voxels, and decreased signal variation in optogenetic fMRI. (A) tSNR maps of optogenetic fMRI data processed without (Standard) and with NORDIC PCA. (B) NORDIC PCA increased tSNR in optogenetic fMRI across different cortical and subcortical brain regions. (C) Group-level ofMRI activation maps illustrating that NORDIC PCA processing induced a larger number of activated voxels compared with Standard processing with the same statistical *t*-thresholds. Gray and blue bars in the top right image indicate the optical fiber location for optogenetic stimulation. (D) Group-level ofMRI BOLD timeseries exhibit a lower standard deviation (gray areas and bar charts) across different brain regions after NORDIC PCA processing. Purple areas indicate the optogenetic stimulation period. SS: somatosensory cortex; Cg: cingulate cortex; SC: superior colliculus; LGN: lateral geniculate nucleus; BF: basal forebrain. Data in (B) and (D) are presented as mean ± S.E.M. across animals; *p < 0.05, **p < 0.01, ***p < 0.005.

ofMRI EPI images showed no apparent morphological change with and without NORDIC PCA (Supplementary Fig. 1A). Overall, it was apparent that NORDIC-processed ofMRI data exhibited a dramatic increase in tSNR when compared with Standard processing without NORDIC PCA. In particular, when examining key ROIs of the basal forebrain circuit as previously defined, we observed a nearly twofold increase in tSNR (*p* < 0.05, corrected multiple paired *t*-test; Fig. 3A-B). Furthermore, the fMRI GLM maps exhibited more activated voxels when using NORDIC PCA compared with Standard processing under the same statistical thresholds (Fig. 3C). In fact, since the basal forebrain is considered the major cholinergic output of the central nervous system with projections to many cortical and subcortical regions (Li et al., 2018), we expected widespread brain activation similar to the NORDIC PCA activation map. To further confirm that NORDIC processing did not compromise spatial specificity, we examined NORDIC and Standard activation maps across various *t*-value thresholds (Supplementary Fig. 2). In doing so, we found that many areas of activation observed in NORDIC maps at strict statistical thresholds also appeared in Standard maps as the statistical thresholds were reduced. We also computed brain-wide percent signal change (PSC) maps for both processing methods (Supplementary Fig. 3). Similar to recent works, Standard and NORDIC processing induced similar PSC values, with minor and focal reductions for NORDIC (Dowdle et al., 2023; Faes et al., 2024; Vizioli et al., 2021). When taken together, it is important to note that elevated *t*-values on NORDIC maps did not colocalize with elevated PSC values. These observations suggest that NORDIC does not notably compromise spatial specificity while increasing the sensitivity of detecting neural activation.

The BOLD timeseries also exhibit a lower standard deviation after the application of NORDIC PCA in various ROIs (*p* < 0.05, corrected multiple paired *t*-test; Fig. 3D). These results indicated that NORDIC PCA not only successfully denoised the ofMRI data, but also stabilized the temporal response of optogenetically evoked brain activity. To further compare NORDIC PCA with similar denoising methods targeting thermal noise, we also computed whole-brain tSNR values for the current ofMRI dataset after MPPCA processing (kernel size = [7 7 7] for both approaches). We found that NORDIC PCA and MPPCA both exhibited a significant improvement over Standard processing, but resulted in nearly identical values at the whole-brain and individual ROI level (Supplementary Fig. 4A-B).

**Fig. 4.**
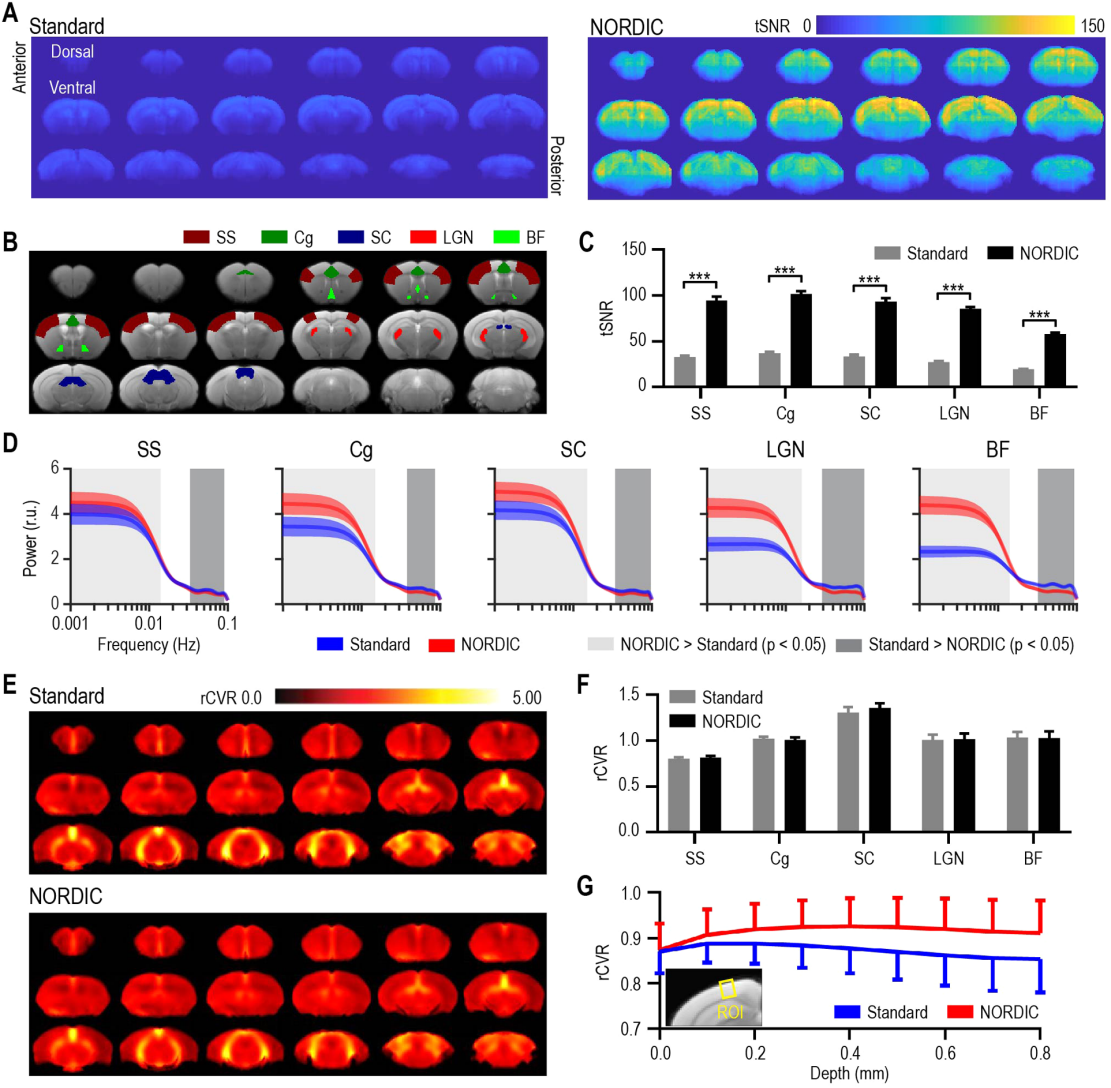
NORDIC PCA increased tSNR and suppressed high-frequency noise in rodent resting-state fMRI, and improved task-free relative cerebrovascular reactivity (rCVR) along cortical layers. (A–C) NORDIC-processed resting-state fMRI data exhibited higher tSNR values across different cortical and subcortical brain regions when compared with Standard processing. (D) When evaluating the group-level power spectra of the BOLD timeseries, all ROIs exhibited increased relative power with NORDIC PCA processing (red curve) compared with Standard processing (blue curve) for frequencies lower than ~0.01 Hz. Similarly, the relative power for NORDIC PCA processing was lower than Standard processing for all ROIs at frequencies higher than ~0.03 Hz. (E–F) Group-level rCVR maps showed no apparent morphological or numerical changes with NORDIC processing at the whole-brain or ROI level. (G) NORDIC PCA significantly increased rCVR along the cortical depth. SS: somatosensory cortex; Cg: cingulate cortex; SC: superior colliculus; LGN: lateral geniculate nucleus; BF: basal forebrain. Data in (C), (D), (F), and (G) are presented as mean ± S.E.M. across animals; r.u.: relative unit. For (C), ***p < 0.005, and for (D) the light gray patch indicates NORDIC > Standard, *p* < 0.05, Bonferroni corrected, and the dark gray patch Standard > NORDIC, *p* < 0.05, Bonferroni corrected.

rsfMRI EPI images also showed no apparent morphological change with and without NORDIC PCA (Supplementary Fig. 1B). NORDIC-processed rsfMRI data revealed a higher tSNR compared with Standard processing without NORDIC PCA by a factor of more than three (*p* < 0.001, corrected multiple paired *t*-test; Fig. 4A-C), indicating that NORDIC PCA was successful at denoising the fMRI data and preserving signal integrity. In fact, such an improvement (>3 times) may enable experiments in which only low SNR and high-resolution fMRI data are available (Vizioli et al., 2021). It is important to note that the tSNR of the rsfMRI data here is lower (for NORDIC and Standard cases) than that of the previously described ofMRI data. This difference in tSNR between the ofMRI and rsfMRI datasets is likely due to differences in (1) the voxel size (0.047 mm^3^ vs. 0.02 mm^3^), (2) the receiver coils used (surface coil vs. Bruker CryoProbe), (3) the receiver bandwidth (3125 Hz vs. 2500 Hz), and (4) the number of frequency- and phase-encoding steps (i.e. matrix size, 64 x 64 vs. 80 x 35). Nevertheless, all of the values reported here fall within normal ranges reported throughout the literature (Edelman et al., 2021; Grandjean et al., 2020; Jung et al., 2019). Despite these differences, we specifically attempted to optimize the raw tSNR values using a relatively low TE (12 ms) (see Section 2.4) (Bhavsar et al., 2014), rather than optimizing BOLD signal change with a higher TE (Han et al., 2019). We chose to optimize SNR to both ensure that the Gaussian assumption of NORDIC PCA holds and to follow recent trends from human NORDIC work that focus on paradigms such as multiband imaging and laminar fMRI that also aim to maximize SNR (Dowdle et al., 2023; Knudsen et al., 2023).

When comparing the power spectra in specific brain areas, we found across all ROIs that NORDIC PCA induced a significantly higher relative power in low frequencies below ~0.01 Hz when compared with Standard processing (Fig. 4D, light gray bars, *p* < 0.05, Bonferroni corrected). Accordingly, we also found in the same ROIs that NORDIC PCA induced a lower relative power in high frequencies greater than ~0.03 Hz (Fig. 4D, dark gray bars, *p* < 0.05, Bonferroni corrected). Interestingly, we found the strongest effects in the BF and LGN, which are much smaller regions than the other ROIs included here. Accordingly, these regions are more susceptible to noise at high spatial resolution, and it is expected that the preserved spatial precision of NORDIC PCA would impact these regions to greater extent than larger ROIs. Other resting-state studies investigating similar infraslow BOLD profiles suggest that a large portion of spontaneous brain activity can be explained by coactivation patterns (CAPs), which are thought to represent fluctuating brain-wide states (Gutierrez-Barragan et al., 2019, 2022). While more general functional connectivity analysis can benefit from frequency content up to 0.20–0.25 Hz using anesthetic protocols similar to that used in the current work (Cabral et al., 2023; Grandjean et al., 2014), CAPs specifically exhibit a dominant frequency content below 0.03 Hz. Therefore, the overall shift that we observe in the content of regional power spectra toward lower frequencies suggests that the application of NORDIC PCA may help to better detect CAP profiles and to identify their time-varying organizing principles.

The average whole-brain rCVR maps and ROI-specific rCVR values exhibited no apparent changes with and without NORDIC PCA (Fig. 4E-F), which support previous studies that report little effect of denoising strategies on rCVR values (Cohen et al., 2021; Moia et al., 2021). Nevertheless, Levene’s F-test showed that NORDIC PCA processing (σ²: 0.0116) induced a significantly lower variance in rCVR values compared with Standard processing (σ²: 0.0144, *p* < 0.05). The lack of whole-brain differences led us to hypothesize that the effect of NORDIC PCA on rCVR may lie at a finer spatial scale than a full anatomical ROI. As such, we examined the rCVR values as a function of cortical depth (not layer specific) and found that Standard processing led to a gradual drop in rCVR values with increasing depth. By contrast, NORDIC PCA preserved nearly constant rCVR values across all depths (*p* < 0.0001, one-way ANOVA; Fig. 4G). Since rCVR is thought to reflect the conditions of capillaries, as they are the primary site of oxygen exchange in the brain, we expect a constant rCVR across all cortical depths as the neocortex consists of a uniform capillary bed (Kirst et al., 2020). In this sense, it appears that rCVR values extracted from NORDIC PCA in the cortex better reflect the underlying physiology (Fig. 4G). With this being said, as rCVR was originally developed and validated in humans, we are applying it here to mouse data as vasodilation is thought to be evolutionarily conserved across these two species. While this assumption is sufficient to begin exploring rCVR in mice, future studies should extend this investigation by validating rCVR through the direct manipulation of CO_2_ content, perhaps by manually changing either the ventilation rate or the amount of CO_2_ being inhaled. Nevertheless, these results add to the growing body of work investigating how rCVR values may be impacted by fMRI signal quality, and suggest that such information examined at a fine spatial scale (i.e. across cortical depth) may in fact be affected by NORDIC PCA and higher tSNR.

**Fig. 5.**
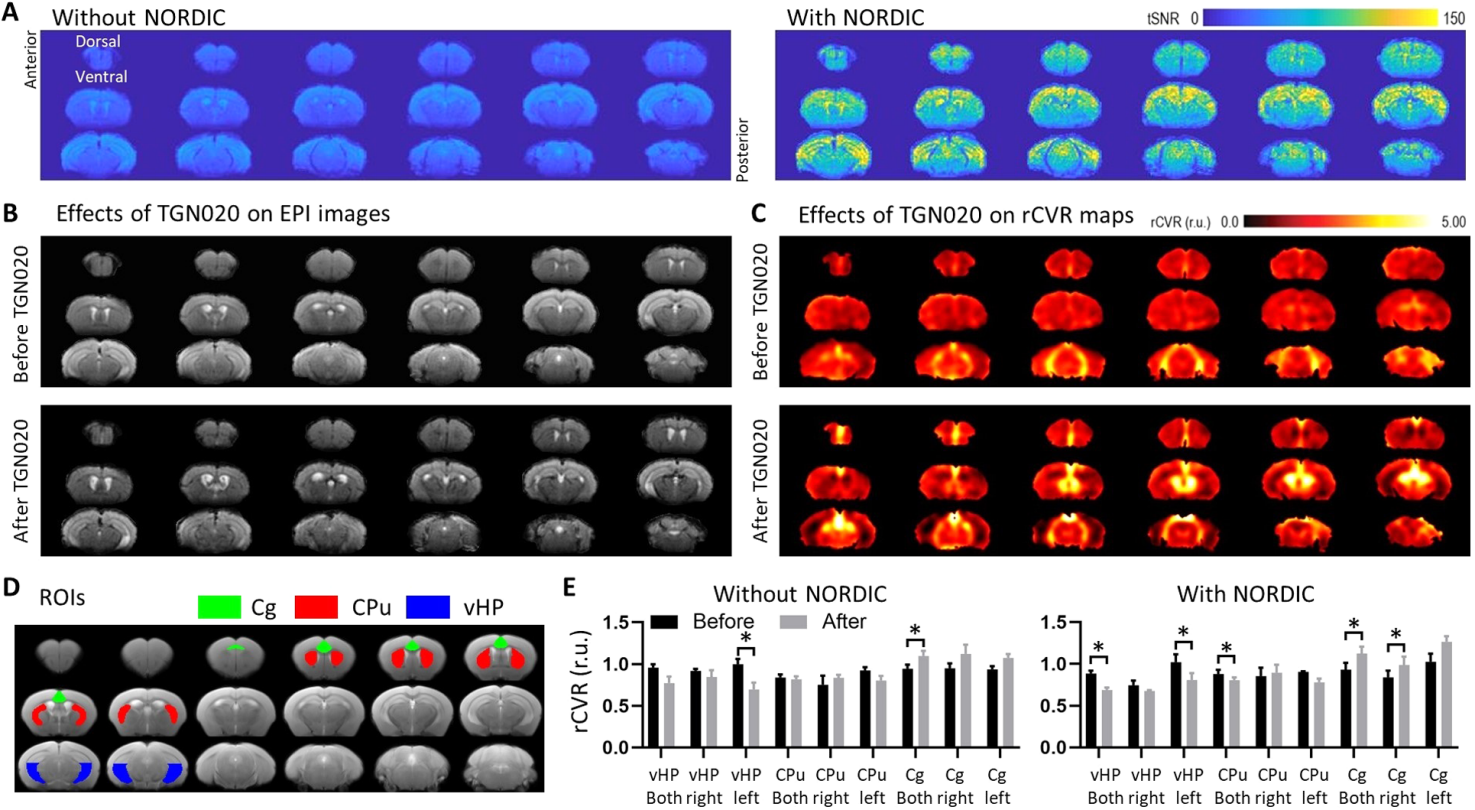
NORDIC PCA improved the detection of TGN020-induced aquaporin-4 inhibition on rCVR. (A) NORDIC-processed resting-state fMRI data for aquaporin-4 inhibition experiments exhibited higher tSNR when compared with Standard processing. (B) NORDIC-processed resting-state fMRI echo-planar imaging (EPI) of a representative mouse before and after intrathecal TGN020 infusion showed no major EPI distortion. (C) rCVR maps of a representative mouse before and after intrathecal TGN020 infusion revealed quantitative rCVR changes without apparent morphological change. (D–E) rCVR significantly decreased in the left vHP and increased in bilateral Cg after intrathecal TGN020 infusion both with and without NORDIC PCA. Furthermore, rCVR significantly decreased in the bilateral vHP and CPu, and increased in the right Cg after intrathecal TGN020 infusion only after NORDIC PCA. These results indicate that NORDIC PCA improved the detection of TGN020-induced aquaporin-4 inhibition on rCVR. CPu: caudate putamen; Cg: cingulate cortex; vHP: ventral hippocampus. Data in (E) are presented as mean ± S.E.M. across animals; r.u.: relative unit; *p < 0.05.

Similar to rsfMRI, the EPI images for the TGN020-induced AQP4 inhibition experiments showed no apparent morphological change with and without the application of NORDIC PCA (Supplementary Fig. 1C). In addition, TGN020 injection did not induce apparent EPI distortions or morphological rCVR changes (Fig. 5A-B). After intrathecal TGN020 infusion, rCVR significantly decreased in the left vHP and increased in the bilateral Cg, both with and without NORDIC PCA (*p* < 0.05, paired *t*-test; Fig. 5C-E). Furthermore, rCVR significantly decreased in bilateral vHP and CPu, and increased in the right Cg after intrathecal TGN020 infusion with NORDIC PCA (*p* < 0.05, paired *t*-test; Fig. 5C-E) but not when analyzed without NORDIC PCA (*p* > 0.05). NORDIC is known to reduce signal variance rather than signal intensity (Vizioli et al., 2021), the latter of which affects rCVR measurements. While signal intensity is not changed by NORDIC (Supplementary Fig. 3), the reduction in variability likely affects the *ΔBOLD/BOLD* term of the rCVR equation, and facilitates the delineation of changes in rCVR as significant. With this being said, these findings highlight the heterogeneity of AQP4 function across the cerebral vasculature, which may explain the dissimilar vulnerability of various brain regions to cerebrovascular diseases.

Moreover, these results were reassuring based on the known mechanism of AQP4 in which interaction with Virchow–Robin space water dynamics describes a physiological model for neurovascular coupling and glymphatic flow (Nakada et al., 2017). Specifically, AQP4 mediates water transfer between astrocytes and pericapillary space, and when TGN020 inhibits this process, water transfer is halted and leads to vasodilation that can be detected using a cerebral blood flow or even BOLD fMRI contrast (Igarashi et al., 2013; Komaki et al., 2020). As rCVR measures the ability of blood vessels to dilate in response to changes in blood flow/volume, we would expect these values to also change in response to TGN020 infusion. Importantly, we observed no difference in the rCVR values in these same ROIs either before or after TGN020 infusion when the data were processed with or without NORDIC PCA (Supplementary Fig. 5). These results support the same conclusion from the rsfMRI dataset (Fig. 4E-F) in that NORDIC PCA does not alter rCVR measurements. Nevertheless, when comparing rCVR values before and after TGN020 infusion, we found that data processed with NORDIC PCA exhibited more significant differences compared with Standard processing (Fig. 5E), suggesting that NORDIC PCA improves the detection of TGN020-induced AQP4 inhibition on rCVR.

### 3.2 NORDIC PCA for human pRF fMRI and rsfMRI

**Fig. 6.**
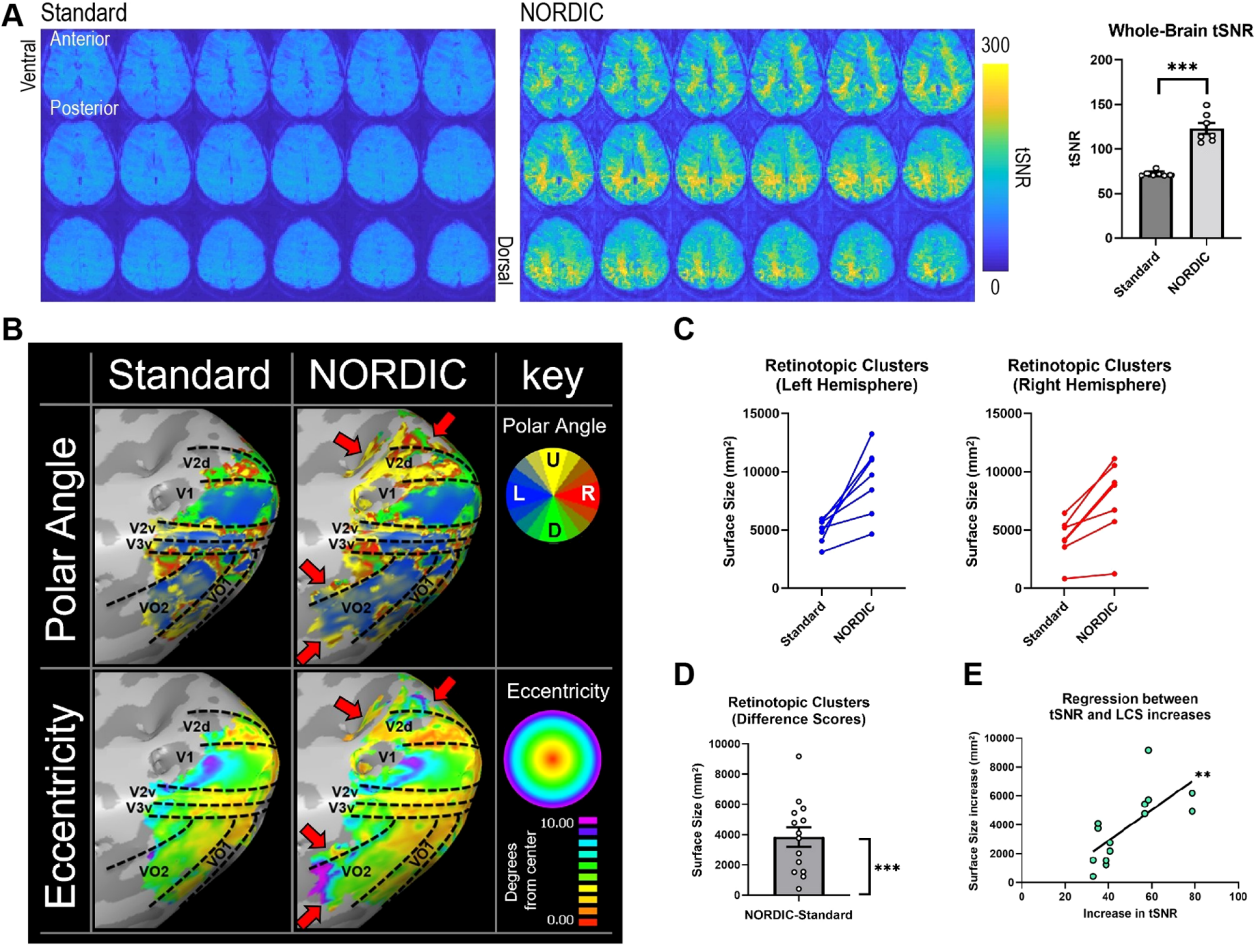
Statistical analyses of task-based fMRI data, examining the effects of NORDIC PCA on tSNR, retinotopic analysis, largest cluster sizes (LCS), and correlation between tSNR and LCS. (A) Whole-brain tSNR values significantly increased with NORDIC PCA compared with Standard processing. (B) Retinotopic cortical organization in a single subject’s right hemisphere for polar angle (top row) and eccentricity (bottom row). Cortical delineation was performed with reference to a previous study (Henriksson et al., 2012). At a default threshold of R > 0.2, the active regions that were shared across Standard and NORDIC-processed data exhibited similar retinotopy. However, NORDIC PCA maps were clearly expanded compared with Standard maps, with more areas/voxels reaching statistical significance at the same statistical threshold (red arrows). U: Up, D: Down, L: Left, R: Right. (C) LCS for Standard and NORDIC-processed data prior to default BrainVoyager preprocessing and pRF mapping are shown for the left and right hemispheres. (D) The LCS difference score (NORDIC minus Standard) was significantly greater than zero. (E) tSNR was significantly correlated with LCS for data processed with NORDIC PCA. Data in (A) and (D) are presented as mean ± S.E.M. across subjects and clusters, respectively; **p < 0.01, ***p < 0.005.

In task-based fMRI using a visual stimulation paradigm for pRF modeling, whole-brain tSNR significantly increased when using NORDIC PCA compared with Standard processing (*t*(6) = 7.784, *p *= 0.000237, d = 3.75), increasing from a mean of 70.33 ± 1.27 to 119.16 ± 6.84 (Fig. 6). Levene’s F-test revealed that this increase was not uniform with a statistically significant increase in tSNR variability following NORDIC processing (*F*(1,12) = 18.49, *p *= 0.001032) (Fig. 6A). Similar to the rodent ofMRI dataset, here we also compared NORDIC PCA with MPPCA (kernel size = [7 7 7] for both approaches) in terms of whole-brain tSNR values. We found that both denoising methods significantly increased global tSNR compared with Standard processing, but exhibited no significant difference between one another (Supplementary Fig. 4C).

fMRI analysis using pRF modeling exhibited similar cortical delineation points in the cortical surface space for both NORDIC and Standard processing methods. Importantly, there was no apparent deterioration or alteration in the resulting retinotopic mapping for regions that reached statistical significance in both cases (Fig. 6B). This indicates that NORDIC PCA preserves the underlying spatiotemporal precision of BOLD signal changes that are necessary for effective retinotopic mapping. Furthermore, compared with Standard processing, NORDIC PCA expanded the cortical regions that reach statistical significance at a consistent statistical threshold (red arrows in Fig. 6B). Importantly, voxels which only exceeded the statistical significance threshold with NORDIC processing routinely showed unique preferential responsivity in terms of polar angle and/or eccentricity when compared with neighboring regions that exceeded the statistical significance threshold in both preprocessing cases. The lack of spatial blurring across an expanded set of significant and neighboring voxels in terms of these characteristics suggests that NORDIC preserves spatial specificity of pRF mapping while also increasing sensitivity (Fernandes et al., 2023). Another recent study examining human tonotopy also found that NORDIC processing preserved tonotopic gradients around the Heshl’s Gyrus (Faes et al., 2024). These comparable effects of NORDIC processing on sensory-based mapping studies further suggest that this approach preserves spatial specificity compared with Standard processing.

The largest cluster sizes (LCS) observed within each hemisphere of the cortical surface space increased with NORDIC PCA for all subjects compared with Standard processing (Fig. 6C). This was further confirmed using the difference scores (NORDIC LCS–“Standard” LCS) which revealed that NORDIC PCA induced a significant increase in LCS, with a mean difference of 3833.57 ± 643.2 voxels (*t*(13) = 5.960, *p *= 0.000047, d = 1.59) (Fig. 6D). Furthermore, the largest LCS observed per hemisphere positively correlated with tSNR increase, as shown using simple linear regression (*F*(1,12) = 11.95, *p *= 0.0048, r^2^ = 0.4989) (Fig. 6E). This indicates that larger increases in tSNR from NORDIC PCA result in larger cluster sizes.

Overall, we show that NORDIC PCA is suitable for pRF modeling by preserving the spatial precision of signals necessary for retinotopic mapping. As a result, more regions with subtle activity changes can be detected with the same or less statistical power to improve specificity. NORDIC PCA can be particularly beneficial for datasets with relatively low tSNR, such as those with a large number of volumes, with single subject data, or with data acquired using low field strengths. This approach may also help to improve the analysis of data already acquired. Taken together, NORDIC PCA exhibits key benefits as a complementary analysis to improving topographical fMRI mapping.

NORDIC-processed human rsfMRI data exhibited a higher tSNR compared with the Standard processing without NORDIC PCA by more than 50% (*p* < 0.005, corrected multiple paired *t*-test; Supplementary Fig. 6A), indicating that NORDIC PCA successfully denoised the data. The average rCVR maps and values exhibited no apparent changes with and without NORDIC PCA for different ROIs that include VC, MC, CPu, HP, and BF (Supplementary Fig. 6B). Since lower signal variance is expected in human rCVR with NORDIC PCA, future clinical studies may benefit from the increased sensitivity of detecting rCVR changes with NORDIC PCA. Similar studies may also leverage the increase in tSNR to push for the acquisition of rsfMRI data with higher resolution than the current clinical standard.

### 3.3 Technical considerations and future directions

As we included both rodent and human fMRI data in the current work, it is crucial to examine whether and how NORDIC PCA affects data from these two species. Overall, we observed a notable increase in tSNR for all contexts across both rodent and human datasets. In fact, for fMRI data involving evoked signals, such as ofMRI in rodents and task-based fMRI in humans, we observed quite a similar percent improvement (~50%) in tSNR values when using NORDIC PCA compared with Standard processing. For rsfMRI, we observed a similar percent improvement for the human dataset; however, in the rodent dataset, this improvement was even more pronounced (>100%). As NORDIC PCA is intended to denoise with preserved spatial precision, it is possible that the impact of this approach is stronger on data with higher spatial resolution. While the human datasets were acquired with similar voxel sizes, the rsfMRI rodent dataset was acquired at over twice the spatial resolution (0.02 mm^3^ voxel size) as the ofMRI dataset (0.047 mm^3^ voxel size). The higher spatial resolution may, therefore, cause an initially low tSNR that leads to an even larger improvement using NORDIC PCA. Future studies may benefit from examining the effects of such a parameter, as well as individual variability and physiological fluctuations, on tSNR improvements with and without NORDIC PCA.

It is also important to note that in the current work, we only applied NORDIC PCA to magnitude images since most fMRI data are stored in magnitude rather than complex form. NORDIC PCA was originally introduced for complex images that exhibit Gaussian noise, since in this context the spectral properties are easily defined. By contrast, magnitude images exhibit Rician noise; however, recent studies have re-emphasized that Rician noise can be treated as Gaussian for even very low levels of SNR (Fernandes et al., 2023). Nevertheless, it is worth noting that denoising with magnitude data increases the point-spread function more than when using complex data; however, we must also consider that the previously reported loss of spatial precision is much less than the blurring induced by other methods (Vizioli et al., 2021). Fortunately, the SNR levels in the current work are high enough that the Gaussian assumption of NORDIC PCA still holds for magnitude images. This is notable when comparing NORDIC PCA with MPPCA, which was tested and optimized for magnitude images (Manzano Patron et al., 2024), and also performs well at high SNR (similar to complex images) (Does et al., 2019). With this being said, magnitude images contain spatially correlated noise, which can further accumulate through the use of multichannel receiver coils (CryoProbe) and partial Fourier acquisition approaches, and must be accounted for. In this regard, one key advantage of NORDIC PCA over MPPCA is that it addresses this confound by mapping the spatially varying and correlated noise to zero mean and spatially identical noise in an individual patch using g-factor normalization (Fernandes et al., 2023; Henriques et al., 2023; Moeller et al., 2021). In fact, this is similar to the Threshold PCA (TPCA) and General PCA (GPCA) approaches proposed in Henriques et al. (2023) that avoid correcting for spatial varying noise by using local noise estimation instead. In such work, the application of TPCA was representative of NORDIC PCA and was shown to be robust across a wide range of conditions that introduce varying amounts of spatial correlations.

The differences between NORDIC PCA and MPPCA on global tSNR were modest for both the rodent ofMRI and human pRF fMRI datasets. This relatively small difference in tSNR values is not entirely unexpected as comparisons using *t*-statistics from GLMs observed similarly subtle improvements with NORDIC PCA compared with MPPCA (Dowdle et al., 2023; Vizioli et al., 2021). Along these lines, we must also acknowledge the potentially suboptimal NORDIC parameters used for such a comparison in the current work. In the majority of the NORDIC results presented here, we used the default 11:1 ratio of spatial to temporal voxels which automatically calculates an optimal kernel size. However, when directly comparing NORDIC and MPPCA, we chose to use a kernel size of [7 7 7] as these values define the previously established parameter space for this family of denoising approaches. However, these values were derived using diffusion MRI datasets with around 100 volumes, whereas fMRI datasets often contain many more. As the latter is the case for datasets included in the current work, it is possible that the fixed kernel size may not be ideal for the various fMRI contexts examined here. Nevertheless, to our knowledge, there has yet to be a systematic investigation of these parameters and we, therefore, utilized default values under the assumption that further optimization would lead to added benefits. Future in-depth work examining how changing NORDIC parameters affects denoising performance would greatly facilitate optimal use throughout the community.

Finally, tSNR should be considered along with other measures of thermal noise reduction such as spatial precision, which has already been extensively compared between NORDIC PCA and MPPCA (Fernandes et al., 2023; Henriques et al., 2023; Moeller et al., 2021). Therefore, while the expansion of NORDIC PCA to magnitude images here demonstrates additional value in its flexibility to improve fMRI data stored in a different and more common format, the datasets consist of relatively small sample sizes and future studies with larger cohorts may more comprehensively clarify the full potential of this method. For example, the application of NORDIC PCA to magnitude data from different preclinical and clinical datasets with low tSNR, such as advanced diffusion MRI modeling (Moeller et al., 2021; Sun et al., 2020), diffusion functional MRI (dfMRI) (Le Bihan et al., 2006), and line-scanning fMRI may help address fundamental biomedical questions, such as elucidating the intrinsic physiological sources of the underlying BOLD mechanisms (Toi et al., 2022).

In conclusion, our results demonstrate that NORDIC PCA can improve the detection of brain activity in both rodent and human fMRI by removing thermal noise, and subsequently increasing tSNR and reducing signal variance. We provide evidence for this improvement across a variety of different fMRI contexts that range from task-based optogenetic stimulations and topological mapping to task-free cerebrovascular reactivity measurements with and without AQP4 inhibition via TGN020 infusion. These findings may in the future play an important role in improving overall fMRI data quality and sensitivity for translational and preclinical neuroimaging research.

## Supplementary Material

Supplementary Material

## Data Availability

The data and codes that support the findings of this study are available from the corresponding authors upon reasonable request.
