# NOise Reduction with DIstribution Corrected (NORDIC) principal component analysis improves brain activity detection across rodent and human functional MRI contexts

**DOI:** 10.1162/imag_a_00325

**Published:** 2024-10-24

**Authors:** Russell W. Chan, Giles Hamilton-Fletcher, Bradley J. Edelman, Muneeb A. Faiq, Thajunnisa A. Sajitha, Steen Moeller, Kevin C. Chan

**Affiliations:** Department of Ophthalmology, New York University Grossman School of Medicine, New York, NY, United States; Neuroscience Institute, New York University Grossman School of Medicine, New York, NY, United States; Tech4Health Institute, New York University Grossman School of Medicine, New York, NY, United States; E-SENSE Innovation & Technology, Hong Kong, China; Hong Kong Centre for Cerebro-cardiovascular Health Engineering (COCHE), Hong Kong, China; Brain-Wide Circuits for Behavior Research Group, Max Planck Institute of Biological Intelligence, Planegg, Germany; Emotion Research Department, Max Planck Institute of Psychiatry, Munich, Germany; Center for Magnetic Resonance Research (CMRR), University of Minnesota, Minneapolis, MN, United States; Department of Radiology, New York University Grossman School of Medicine, New York, NY, United States; Department of Biomedical Engineering, Tandon School of Engineering, New York University, New York, NY, United States; Department of Ophthalmology, School of Medicine, University of Pittsburgh, Pittsburgh, PA, United States

**Keywords:** NORDIC, optogenetic fMRI, resting-state fMRI, population receptive field, cerebrovascular reactivity, thermal noise

## Abstract

NOise Reduction with DIstribution Corrected (NORDIC) principal component analysis(PCA) has been shown to selectively suppress thermal noise and improve thetemporal signal-to-noise ratio (tSNR) in human functional magnetic resonanceimaging (fMRI). However, the feasibility to improve data quality for rodent fMRIusing NORDIC PCA remains uncertain. NORDIC PCA may also be particularlybeneficial for improving topological brain mapping, as conventional mappingrequires precise spatiotemporal signals from large datasets (ideally ~1 houracquisition) for individual representations. In this study, we evaluated theeffects of NORDIC PCA compared with “Standard” processing invarious rodent fMRI contexts that range from task-evoked optogenetic fMRI toresting-state fMRI. We also evaluated the effects of NORDIC PCA on humanresting-state and retinotopic mapping fMRI via population receptive field (pRF)modeling. In rodent optogenetic fMRI, apart from doubling the tSNR, NORDIC PCAresulted in a larger number of activated voxels and a significant decrease inthe variance of evoked brain responses without altering brain morphology. Inrodent resting-state fMRI, we found that NORDIC PCA induced a nearly threefoldincrease in tSNR and preserved task-free relative cerebrovascular reactivity(rCVR) across cortical depth. NORDIC PCA further improved the detection ofTGN020-induced aquaporin-4 inhibition on rCVR compared with Standard processingwithout NORDIC PCA. NORDIC PCA also increased the tSNR for both humanresting-state and pRF fMRI, and for the latter also increased activation clustersizes while retaining retinotopic organization. This suggests that NORDIC PCApreserves the spatiotemporal precision of fMRI signals needed for pRF analysis,and effectively captures small activity changes with high sensitivity. Takentogether, these results broadly demonstrate the value of NORDIC PCA for theenhanced detection of neural dynamics across various rodent and human fMRIcontexts. This can in turn play an important role in improving fMRI imagequality and sensitivity for translational and preclinical neuroimagingresearch.

## 1 Introduction

Functional magnetic resonance imaging (fMRI) is an indispensable tool fornoninvasively mapping task-based brain activity (Bandettini et al., 1992;Kwong et al.,1992;Ogawa et al., 1992),resting-state functional connectivity (Van Essen& Ugurbil, 2012), and cerebrovascular reactivity (Chan et al., 2021;Liu etal., 2020) in humans. In preclinical settings, rodent fMRI is alsovaluable for translational and basic research, such as with using resting-state fMRI(rsfMRI) to identify changes in intrinsic brain-wide networks in disease models(van der Merwe et al., 2021). Rodent fMRIalso provides comprehensive information to determine the globalblood-oxygenation-level-dependent (BOLD) activation patterns in response to themanipulation of neural circuits using optogenetic fMRI (ofMRI) (Chan et al., 2017;Lee etal., 2010;Lin et al., 2016).Despite this utility, fMRI can suffer from a low signal-to-noise ratio (SNR), whichpresents a barrier for identifying statistically significant results. This isusually compounded by limitations in the quantity and quality of data acquisition,for example, when group-level analysis is composed of only a few animals, whenexperimental parameters affect the duration of individual scans, or when lower fieldstrength MRI leads to noisier data. These limitations impede researchers’ability to obtain high SNR data and may require substantial resources, such asadditional animals and cost, to resolve.

One recent approach to improving the quality of rodent fMRI is communitystandardization, which is already common for clinical data acquisition, but has yetto be fully adopted in preclinical research. Despite differences in individual MRscanners, collaborative efforts can help identify and disseminate optimalacquisition parameters to many researchers across the field (Grandjean et al., 2023). An adjacent trend has alsopushed for the standardization of fMRI data analysis pipelines for human (e.g.fMRIPrep) (Esteban et al., 2019), rodent(e.g. RABIES) (Desrosiers-Gregoire et al.,2022;Grandjean et al., 2020), andeven nonhuman primate (e.g. Pypreclin) (Tasserie etal., 2020) research. These and other pipelines heavily focus onstandardizing basic preprocessing principles such as slice-timing correction, headmotion estimation, susceptibility distortion correction, and atlas/templateregistration, and often provide concise quality control reports. While these stepsaccount for a large portion of the necessary analysis and are more or less similaracross various rodent pipelines, there is a lack of consensus on other denoising andconfound correction techniques. For example, techniques based on independentcomponent analysis (e.g. ICA-AROMA, ICA-FIX) (Pruimet al., 2015;Zerbi et al., 2015)have been effectively utilized to correct vascular and/or motion-related noise.Additional nuisance regression approaches using signals from nonbrain regions havealso proven effective at reducing additional sources of physiological noise (e.g.CompCor, GLMdenoise) (Behzadi et al., 2007;Chuang et al., 2019;Kay et al., 2013). However, although these algorithmshave effectively increased the SNR of fMRI datasets, thermal noise is still largelyoverlooked in most pipelines.

Recently, NOise Reduction with DIstribution Corrected (NORDIC) principal componentanalysis (PCA) has demonstrated an ability to selectively suppress thermal noise andto significantly improve temporal SNR (tSNR) in human diffusion tensor imaging(Moeller et al., 2021) and fMRI (Vizioli et al., 2021). NORDIC PCA isconstructed to specifically target Gaussian noise induced by the thermal sources inMRI system electronics, while leaving intact other non-Gaussian noise caused byneuronal fluctuations, respiration, etc. (Moeller etal., 2021;Vizioli et al., 2021).Other PCA approaches have been developed to separate these signal subspaces (Behzadi et al., 2007;Tierney et al., 2016); however, they often require asubjective definition of components that belong to each category and can, therefore,produce results that vary greatly across researchers and laboratories. Thislimitation was largely accounted for with the implementation of Marchenko-Pastur PCA(MPPCA) (Veraart et al., 2016). This approachprovides an objective threshold of components based on the right edge of theMarchenko-Pastur distribution, which is a universal signature of noise in samplecovariance matrices. Nevertheless, this approach has also been shown to lead tospatial blurring, which is undesirable in a field that continues to push forincreasingly high spatial resolution data (Fernandeset al., 2023;Moeller et al.,2021). By contrast, NORDIC PCA also identifies an objective, data-driventhreshold for the number of components to remove, but is specifically tuned topreserve spatial precision. As such, NORDIC PCA is a particularly promising thermalnoise reduction technique that can complement other fMRI analysis pipelines. WhileNORDIC PCA has been tested and verified in a number of diverse human MRI contexts,the utility of this technique to improve various aspects of rodent fMRI analysis hasyet to be extensively evaluated. Based on previous human fMRI results, we predictthat the tSNR can be improved for rodent fMRI; however, it is currently unknownwhether NORDIC PCA can also improve fMRI data quality for additional questionsincluding but not limited to cerebrovascular reactivity and glymphatic systemfunction.

In this study, we evaluated the effect of NORDIC PCA in an fMRI analysis pipeline fora range of applications that include rodent ofMRI and rsfMRI, as well as humanreceptive field mapping fMRI and task-free rsfMRI. We first evaluated theapplicability of NORDIC PCA to ofMRI by quantifying the change in tSNR values, andby examining the overall effect on brain-wide BOLD activation maps. For rodentrsfMRI, we examined the effects of NORDIC PCA on resting-state BOLD signalcharacteristics such as tSNR and relative frequency content. As it has beensuggested that tSNR and signal quality may be linked with relative cerebrovascularreactivity (rCVR) (Chu et al., 2018;Cohen et al., 2021) specifically by reducingsignal variability (Vizioli et al., 2021), wealso examined the effect of NORDIC PCA on task-free rCVR in different brain regions(Chan et al., 2021) and along corticaldepth. Since neurovascular coupling can be altered by factors such as aquaporin-4(AQP4) (Komaki et al., 2020;Nakada et al., 2017), we further investigated the effectof AQP4 inhibition on rCVR by measuring rsfMRI signals before and after intrathecalinfusion of TGN020 (Vandebroek & Yasui,2020), both with and without NORDIC PCA processing. Aside from rodentfMRI, we also applied NORDIC PCA to human population receptive field (pRF) mappingand rsfMRI data. Using human fMRI data can both verify the analysis pipelineutilized here for rodent fMRI and enable a within-study comparison with rodentresults. This analysis also extends the evaluation of NORDIC PCA to additional humanapplications that may benefit from denoising techniques that preserve spatialprecision. To address these aims, we evaluated the effect of NORDIC PCA on tSNR,retinotopic cluster size, and cortical organization, as well as on task-free rCVR indifferent brain regions. Overall, the results of this study can help improve fMRIimage quality and sensitivity for translational and preclinical research.

## 2 Materials and Methods

### 2.1 Animal inclusion and preclinical study approval

Adult male ChAT-IRES-Cre knock-in mice (n = 4, 8–10 weeks old; TheJackson Laboratory: Strain #006410) and C57BL/6J mice (cortical depth analysis n= 13, 12–16 weeks old; AQP4 inhibition n = 3, 16 weeks old;The Jackson Laboratory: Strain #000664) were used for our ofMRI and rsfMRIexperiments, respectively. Animals were housed under a 12-hour light/dark cyclewith access to food and water*ad libitum*. All experimentalprocedures and animal husbandry were performed in strict accordance with theU.S. National Institutes of Health and New York University Grossman School ofMedicine Institutional Animal Care and Use Committee guidelines.

### 2.2 Stereotaxic virus injection for ofMRI experiments

**Fig. 1.**
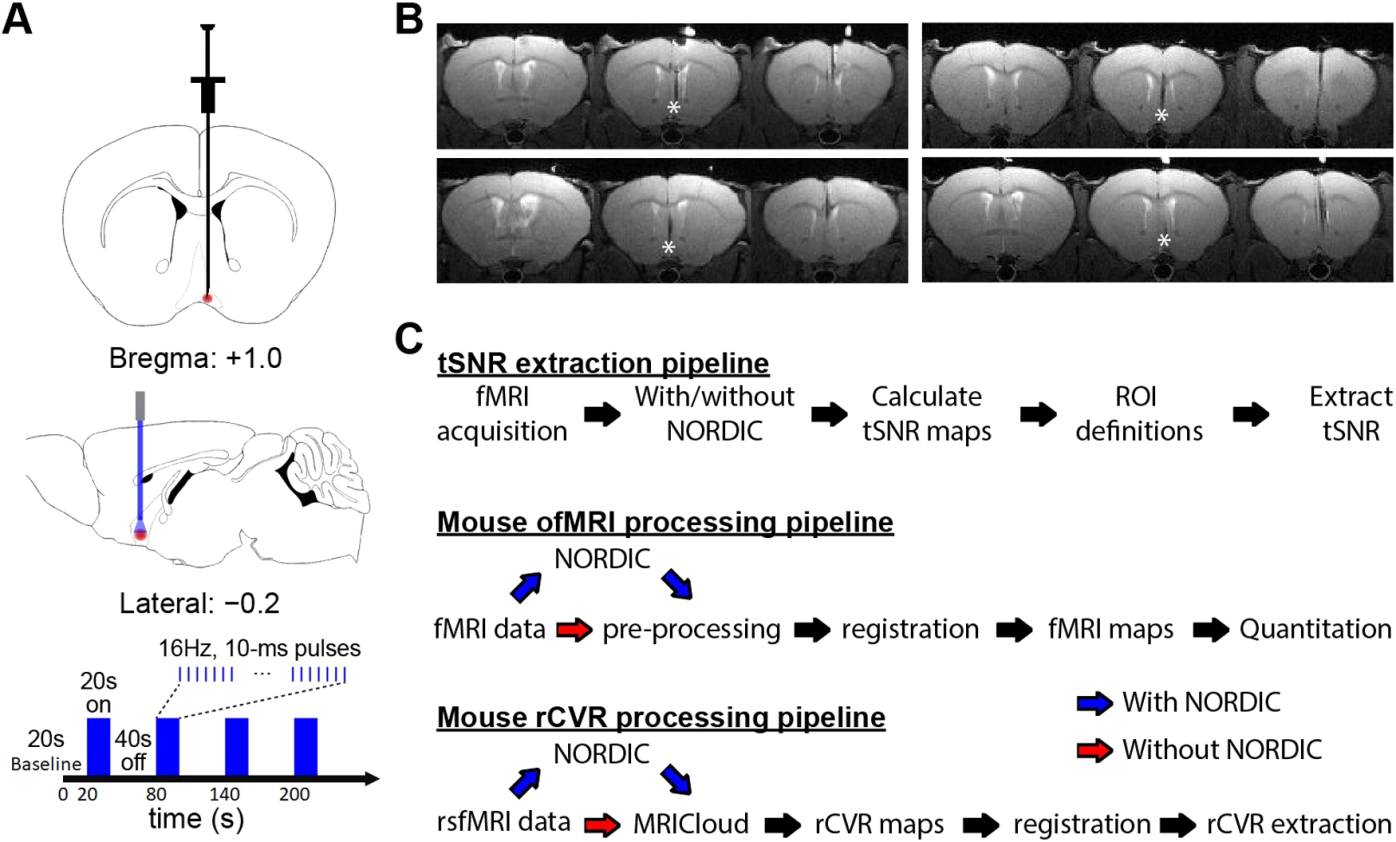
Viral injection, fiber implantation, and block-design stimulation foroptogenetic functional fMRI (ofMRI) enable mapping of brain-widecholinergic basal forebrain networks, and processing pipelines toevaluate the effects of NORDIC-correction in mouse ofMRI andresting-state functional MRI (rsfMRI)-derived rCVR. (A) 1.0 μL ofthe AAV5/DIO-ChR2–EYFP virus was injected to the basal forebrain(+ 1.0 mm AP, − 0.2 mm ML, + 5.2 mm DV) at a 50nL/min flow rate, after which a custom-designed fiber optic cannula wasimplanted. After at least 4 weeks, optogenetic fMRI experiments wereperformed using a block-design stimulus consisting of blue light (473nm) at 16 Hz. Each run consisted of a 20 sec baseline, followed by 4blocks with 20 sec of stimulation and 40 sec of subsequent rest. (B)Cannula locations were validated in all animals used for the optogeneticfMRI experiments with T2-weighted structural MRI. * indicates thelocation of the fiber tip. (C) Processing pipelines used to evaluate theeffects of NORDIC PCA on tSNR (top), ofMRI activation (middle), andrsfMRI-derived rCVR (bottom).

For ofMRI experiments (Chan, Cron, et al.,2022;Chan, Lee, et al., 2022;Chan et al., 2017;Lee et al., 2010), a double-floxed inverted openreading frame (DIO) recombinant adeno-associated virus-5 (AAV5) was used toexpress ChR2-EYFP in Cre-expressing neurons. The recombinant AAV vector waspackaged by the University of North Carolina viral vector core at a titer of 4× 10^12^particles/mL. Animals were anesthetized with isoflurane(induction 3%, maintenance 1.5%–2%) and secured in a stereotactic framewith nonrupturing ear bars. A heating pad was used to maintain body temperatureat 37°C, and sterile ocular lubricant was applied to the eyes to preventdesiccation during surgery. Buprenorphine (0.1 mg/kg) was injectedsubcutaneously for analgesia. After a midline incision in the scalp, a smallcraniotomy was created using a dental drill, followed by virus injection andcannula implantation in the basal forebrain (+ 1.0 mm AP, − 0.2 mmML, and + 5.2 mm DV, according to the Allen Brain Atlas:https://www.brain-map.org;Fig. 1A). Then, 1.0 μL of theAAV5/DIO-ChR2–EYFP virus was injected at 50 nL/min, driven by amicrosyringe pump controller. After injection, the needle was held in place for10 min before slowly being retracted from the brain. A custom-designed 200μm diameter fiber optic cannula was mounted and secured on the skullusing light-cured dental cement (Kuraray Inc.), with the optical fiber extendingfrom the cannula’s base to the desired depth at 0.1 mm above theinjection site (Fig. 1A). Followingsurgery, mice were kept on a heating pad until recovery from anesthesia and weregiven carprofen (5 mg/kg, subcutaneously) daily for 2 days as postoperativeanalgesia to minimize discomfort. All fMRI experiments were conducted at least 4weeks following virus injection to ensure proper ChR2 expression. Optical fiberlocation was validated in all animals used for ofMRI experiments by examiningT2-weighted structural MRI images (Fig.1B).

### 2.3 Intrathecal surgery for rsfMRI of aquaporin-4 inhibition

For AQP4 inhibition rsfMRI experiments, a polyethylene tubing (inner diameter= 0.28 mm; outer diameter = 0.60 mm) was surgically placedintrathecally at the lumbar region (L4–L5) of healthy adult mice. TGN020(30 mg/kg) was infused at a rate of 1.6 μL/min for 30 min.

### 2.4 MRI acquisition—rodent ofMRI and rsfMRI

All rodent MRI experiments were carried out on a 7-Tesla Bruker Biospec (70/30)small animal MRI system at the Preclinical Imaging Laboratory core facility atNew York University Grossman School of Medicine using a transmit-only birdcagecoil in combination with (1) a custom-designed actively decoupled 1 cm diameterreceive-only surface coil for ofMRI experiments or (2) a Bruker 1 H four-channelphased array receive-only CryoProbe for rsfMRI experiments (half-shell innerdiameter = 20 mm; length = 85 mm). Animals were initiallyanesthetized in an induction chamber with 3% isoflurane and were provided aninitial subcutaneous (s.c.) bolus of 0.1 mg/kg dexmedetomidine before beingplaced onto the MRI-compatible cradle with ears, teeth, and head secured. Tomaximize SNR, the receiver coil was positioned on top of the head and centeredover the brain, after which the animal was placed into the isocenter of themagnet. Throughout scanning, animals were lightly anesthetized and sedated usinga combination of low-dose isoflurane (0.25% isoflurane) and dexmedetomidine(continuous s.c. infusion at 0.1 mg/kg/hour). During all experiments, animaltemperature was maintained throughout fMRI acquisition using a heatedcirculating water bath. Continuous physiological monitoring was also performedusing an MRI-compatible system (SA Instruments). Vital signs were within normalphysiological ranges (rectal temperature: 36.5–37.5°C, heart rate:260–420 beat/min, breathing rate: 80–120 breath/min, and oxygensaturation: >90%) throughout the duration of the experiments. Heart ratewas stable for individuals and only varied across animals, but in general waswithin the same range that has been reported elsewhere (You et al., 2021).

For ofMRI, 20 contiguous 0.75 mm thick coronal slices were positioned to coverthe whole brain. T2-weighted high-resolution anatomical images were acquiredprior to ofMRI to check for brain damage and to confirm accurate fiber location.These anatomical images were acquired using a rapid acquisition with relaxationenhancement (RARE) sequence with RARE factor = 4, echo time(TE)/repetition time (TR) = 8.3/3000 ms, field of view (FOV) = 16x 16 mm^2^, and 160 x 160 matrix size. ofMRI measurements were obtainedfor the same slice locations using a single-shot gradient-echoecho-planar-imaging (GE-EPI) sequence with TE/TR = 12/1000 ms, FOV= 16 x 16 mm^2^, 64 x 64 matrix size, voxel size (VOX) =0.25 x 0.25 x 0.75 mm^3^, receiver bandwidth = 3125 Hz, andnumber of repetitions = 260. To reduce acquisition time, we utilizedpartial Fourier k-space-encoding acceleration in the phase-encoding dimensionusing an acceleration factor = 1.2, overscans = 12, and in thefrequency-encoding dimension using an acceleration factor = 1.0 andoverscans = 40.

For rsfMRI, 30 contiguous 0.5 mm thick coronal slices were positioned to coverthe whole brain. T2-weighted high-resolution anatomical images were acquiredprior to rsfMRI as an anatomical reference. These anatomical images wereacquired using a RARE sequence with RARE factor = 4, TE/TR =8.3/3750 ms, FOV = 16×16 mm^2^, and 160 × 160matrix size. rsfMRI measurements were obtained for the same slice localizationsusing a single-shot GE-EPI sequence with TE/ TR = 12/1000 ms, FOV= 16 x 7 mm^2^, 80 x 35 matrix size, VOX = 0.20 x 0.20 x0.50 mm^3^, receiver bandwidth = 2500 Hz, and number ofrepetitions = 600. These rsfMRI data were acquired with the same partialFourier k-space-encoding acceleration as was performed for the ofMRIdataset.

For ofMRI experiments, the MR scanner and laser for optogenetic stimulation weresynchronized using an Arduino Uno with custom codes. The light delivery systemwas maintained outside of the scanner room and long optical cables (<5 m)were used to deliver light into the implanted fibers of the mouse in the scannerbore. Blue light was delivered using a 473 nm DPSS laser that was calibratedprior to scanning to deliver ~1.5 mW at the fiber tip (200 μm).Optogenetic stimulation was delivered at 16 Hz with a pulse width of 10 ms(i.e., 16% duty cycle), as these parameters have previously been shown torobustly activate the cholinergic basal forebrain circuit (Xu et al., 2015). A block-design paradigm wasemployed that began with a 20 sec baseline and was then followed by four 20-secstimulation periods that repeated every 60 sec (40 sec nonlight period) (Fig. 1A).

For AQP4 inhibition rsfMRI experiments, animals were scanned immediately beforeand 30 min after intrathecal infusion of the AQP4 inhibitor TGN020.

### 2.5 Data analysis—rodent ofMRI and rsfMRI

Standard preprocessing using SPM12 (www.fil.ion.ucl.ac.uk/spm) was applied to raw ofMRI and rsfMRImagnitude images with NORDIC PCA or without (Standard) (github.com/SteenMoeller/NORDIC_Raw). In both cases, the first 10brain volumes (dummy volumes) were removed from each dataset prior to additionalprocessing steps to allow the magnetic field to reach equilibrium. The NORDICPCA algorithm for magnitude images follows the same concept as for compleximages, that is, noise flattening followed by spatially invariant noise-basedthresholding using an 11:1 ratio of spatial to temporal voxels. Previous workusing numerical simulations, diffusion MRI and fMRI, has found this ratio to beoptimal (Moeller et al., 2021;Vizioli et al., 2021). This ratio led to akernel size of [14 14 14] and [19 19 19] for the ofMRI and rsfMRI datasets,respectively. In a separate analysis, we also directly compared NORDIC and MPPCAusing the same kernel size. For this we used a kernel size of [7 7 7] for bothmethods as this is the established parameter space for MPPCA. The spatial noisein the images was flattened using a spatial noise estimate with theMarchenko-Pastur distribution asymptotic properties. The threshold in NORDIC PCApatch-based denoising for magnitude images was set proportional to the largestsingular value from a Gaussian distribution for a patch of equal size. Patchoverlapping and averaging with 1/2 the patch size shift between patches wereused. After NORDIC PCA, fMRI image timestamps were adjusted to account for slicetiming differences. This was necessary since for a given time point (i.e. asingle repetition or TR), slices were acquired one after another rather thansimultaneously. Brain extraction was performed using the Amira software (ThermoFisher Scientific). Standard and NORDIC-processed data were then motioncorrected using a 6-parameter rigid registration procedure and smoothed using aGaussian kernel with full width at half maximum of 2 voxels to remove localsources of white noise. Data were linearly detrended to minimize baseline driftcaused by system instability. Briefly, a global linear trend was firstcalculated using linear regression of the temporal signal obtained from thewhole brain. Then, this global linear trend was subtracted from the temporalsignal of each voxel. fMRI data were then temporally band-pass filtered (ofMRI:0.001–0.25 Hz, rsfMRI: 0.001–0.1 Hz) to reduce physiologicalnoise. T2-weighted images from each animal were registered to a custom-madebrain template acquired with the same settings. As inbred mouse lines such asthose used in the current work exhibit a high level of anatomical consistency(Badea et al., 2007;Kovačević et al., 2005), registrationwas performed using an affine transformation (Chan, Cron, et al., 2022;Chan, Lee,et al., 2022;Chan et al.,2017;Lee et al., 2010). Thequality of the registration was performed manually to ensure that there were nomajor errors in alignment.

Whole-brain tSNR maps were calculated by dividing voxel-wise mean by voxel-wisestandard deviation of the BOLD timeseries. In addition, focal tSNR values wereextracted from anatomical regions of interest (ROIs) defined across cortical andsubcortical areas according to the Allen Brain Atlas. These regions include thesomatosensory cortex (SS), cingulate cortex (Cg), superior colliculus (SC),lateral geniculate nucleus (LGN), and basal forebrain (BF).

For ofMRI, a double gamma basis set was convolved with the stimulation blockdesign and a fixed-effects general linear model (GLM) was used to produceactivation maps. To account for the sharper hemodynamic response characterizedin rodents (Lambers et al., 2020;Pisauro et al., 2013;You et al., 2021), we utilized a hemodynamic responsefunction (HRF) with the following values: delay of response = 3.7 sec,delay of undershoot = 4.45 sec, dispersion of response = 0.5 sec,dispersion of undershoot = 0.5 sec, ratio of response undershoot =1.5 sec, onset = 0 sec, and kernel length = 32 sec. Qualitycontrol was performed by manually assessing the GLM results from each animalbefore being included in the group-level analysis. BOLD timeseries wereextracted from predefined anatomical areas by averaging the timeseries of allvoxels within the ROI. The standard deviation of the BOLD timeseries was alsoextracted from each ROI. For rsfMRI, the power spectrum of the BOLD timeserieswas extracted from each predefined ROI.

MRICloud (mricloud.org/) was used togenerate the whole-brain rCVR maps from the rsfMRI data (Chan et al., 2021;Chan, Lee, et al., 2022;Liu et al.,2017,^2022^;Lu et al., 2014). Specifically, the CVR-MRICloud toolaccepts raw ANALYZE files as an input and applies a default processing pipelinethat includes motion correction, spatial smoothing, linear detrending, andtemporal filtering (Liu et al., 2017,^2022^). As such, we used CVR-MRICloudon raw data with and without the application of NORDIC PCA (Fig. 1C). The voxel-wise rCVR index(*α*) was first computed using a GLM containing thenormalized BOLD timeseries (*ΔBOLD/BOLD*) and the globalsignal timeseries (*GS*).*ΔBOLD/BOLD*wascomputed according to the following equation:

$$\frac{\Delta BOLD}{BOLD}=\left(s-mean\left(s\right)\right)/\left(\frac{2\left|s-mean\left(s\right)\right|}{\sqrt{N)}}\right),$$

where*s*is the detrended and filtered BOLD timeseries and*N*is the total number of dynamics. This rescaling ensuresthat the timeseries has a zero mean and norm of$\sqrt{N}/2$(Liu et al., 2022). Voxel-wise rCVR valueswere then obtained by normalizing*α*by the tissue signalintensity averaged across the whole brain (*SI*). These steps canbe summarized as follows (note the residual term*β*wasnot used in this analysis):

$$\begin{array}{l} rCVR=\frac{\alpha }{SI},where\;\alpha \;is\;obtained\;from\;\frac{\Delta BOLD}{BOLD}\\ \;\;\;\;\;\;\;\;\;\;\;\;\;=\alpha \cdot GS+\beta . \end{array}$$

Anatomically constrained rCVR values were extracted using the same previouslydescribed ROIs as well as along the cortical depth, as defined by the AllenBrain Atlas.

### 2.6 Clinical study approval and human subject recruitment

Healthy human subjects were recruited for both pRF fMRI (n = 7,48–70 years old) and rsfMRI (n = 21, 41–79 years old). Theinstitutional review board and ethics committee of the University of Pittsburghapproved this study and all subjects provided written informed consent. Thisstudy followed the tenets of the Declaration of Helsinki and was conducted incompliance with the Health Insurance Portability and Accountability Act.

### 2.7 MRI acquisition—human pRF fMRI and rsfMRI

Human MRI experiments were performed on a 3-Tesla Allegra head scanner (Siemens,Erlangen, Germany) at the Neuroscience Imaging Center at the University ofPittsburgh. Initially, a conventional whole-brain T1-weighted anatomical MRI wasacquired using a 3D MPRAGE pulse sequence with 176 contiguous 1.0 mm thicksagittal slices, TE/TR = 2.5/1400 ms, inversion time(TI) = 800 ms, flipangle = 8°, FOV = 25.6 x25.6 x 17.6 cm^3^, 256 x 256 matrix size,and VOX = 1.0 x 1.0 x 1.0 mm^3^.

**Fig. 2.**
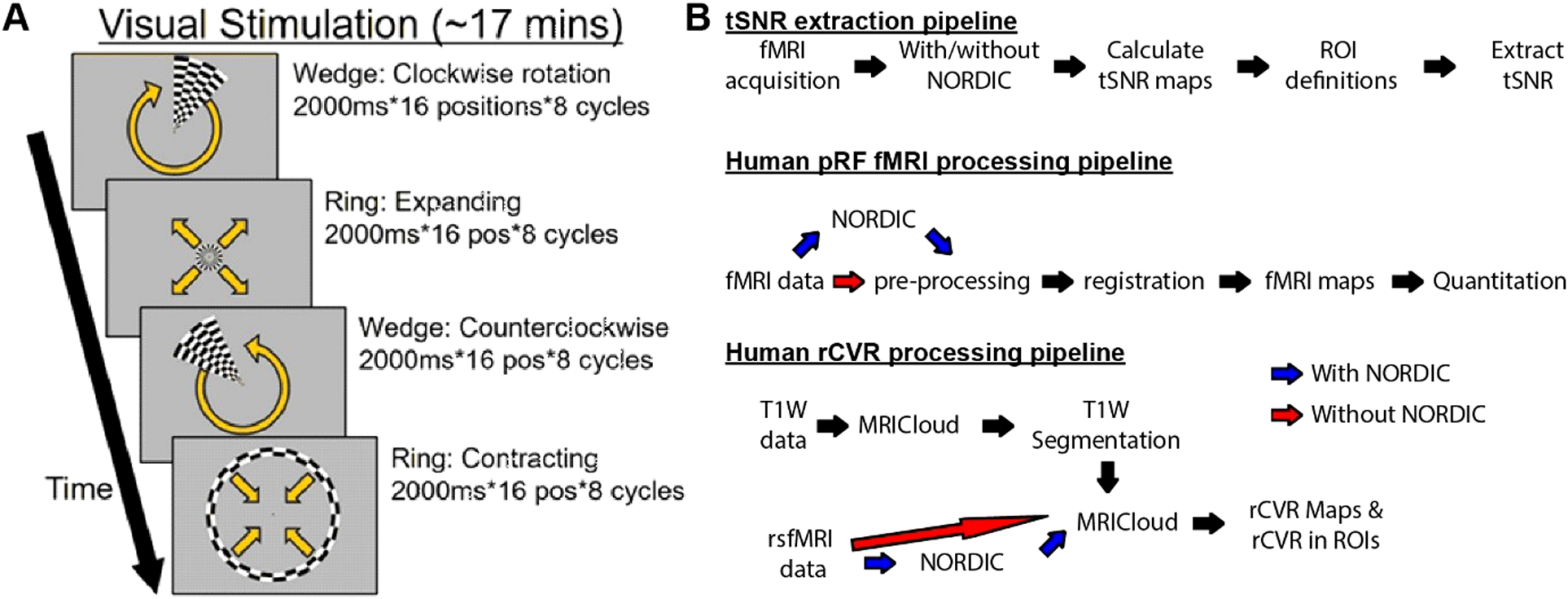
Visual stimulation during task-based fMRI experiments for retinotopicmapping via population receptive field modeling, and processingpipelines used to evaluate the effects of NORDIC PCA in human fMRI. (A)Subjects positioned inside the MRI scanner observed ~17 min of visualstimulation, consisting of four conditions that involved either a movingcheckerboard ring moving clockwise/counter-clockwise or a wedgeexpanded/contracted, respectively (yellow arrows). These four conditionsconsisted of single image presentations lasting 2000 ms, sequentiallymoving across 16 positions and repeating 8 cycles per condition (1024sec total). Visual stimulation was presented on a screen inside thescanner that subtended a 26° visual angle. Subjects were taskedwith responding using a key press on a provided keypad when a centralfixation dot changed color. (B) Processing pipelines used to evaluatethe effects of NORDIC PCA on tSNR (top), pRF fMRI (middle), andrsfMRI-derived rCVR (bottom).

For task-based pRF fMRI, T2*-weighted whole-brain BOLD images werecollected using a single-shot GE-EPI pulse sequence with 28 contiguous 3.24 mmthick axial slices, TE/TR = 26/2000 ms,FOV = 20.5 x 20.5 cm^2^(positioned to ensurecoverage of the bilateral visual cortices), 104 x 104 matrix size,VOX = 2.0 x 2.0 mm^2^, and number of repetitions = 512.During T2*-weighted fMRI scans, subjects underwent retinotopicstimulation via visual presentation of checkerboard rings and wedges on a screenwith a 26° field of view. Subjects were asked to fixate on the center ofthe image and respond with a key press when a small central dot changed. Duringthis procedure, subjects were presented with four different conditions: (1)wedge rotating clockwise, (2) ring expanding, (3) wedge rotatingcounter-clockwise, and (4) ring contracting. Each of these conditions involveddisplaying an image for 2 sec, with 16 positions per repetition and 8repetitions per scan (total scan length = 17 min and 4 sec) (Fig. 2A).

rsfMRI was performed while the participant remained at rest with his/her eyesclosed. T2*-weighted BOLD images were collected using a single-shotGE-EPI pulse sequence covering the whole brain with 38 contiguous 3 mm thickaxial slices, TE/TR = 26/2000 ms,FOV = 20.5 x 20.5 cm^2^, 64 x 64 matrix size, VOX= 2.0 x 2.0 x 3.0 mm^2^, and number of repetitions =240.

### 2.8 Data analysis—human pRF fMRI and rsfMRI

Similar to the rodent fMRI data analysis, whole-brain tSNR maps were calculatedfrom the human pRF fMRI and rsfMRI data with and without the application ofNORDIC PCA by dividing the voxel-wise mean by the voxel-wise standard deviationof the BOLD timeseries (Fig. 2B). NORDICPCA was only applied to magnitude images with the same default settings as wasused for the rodent fMRI datasets (github.com/SteenMoeller/NORDIC_Raw). In usingthe default 11:1 ratio of spatial to temporal voxels, the kernel size for thepRF fMRI and rsfMRI datasets was [18 18 18] and [14 14 14], respectively. Aswith the rodent fMRI analysis, we also compared NORDIC and MPPCA in a separateanalysis using a kernel size of [7 7 7] for both methods.

For pRF maps, fMRI volumes were initially processed either without(“Standard”) or with NORDIC PCA. The Standard and NORDIC-processedimages were then preprocessed by applying brain extraction, slice-timingcorrection, and 3D motion correction. Temporal filtering was not applied to pRFdata as this processing step has previously been shown to artificially increasepRF estimates and bias the interpretation of spatial tuning properties (Morgan & Schwarzkopf, 2020). Thiswas followed by coregistration with the corresponding T1-weighted anatomicalimage, and normalization into the Montreal Neurological Institute (MNI) spaceusing BrainVoyager’s default processing pipeline. The pRF analysis wasperformed according to the method described byDumoulin and Wandell (2008)and implemented in the BrainVoyagersoftware using default parameter settings. Briefly, the pRF mapping proceduremodeled the expected hemodynamic response profile for receptive fields atdifferent locations and sizes in the visual field during the presented visualstimulation. Individual GLMs were computed for every combination of thefollowing parameters: 30 horizontal locations, 30 vertical locations, and 30sizes (0.20–7.00°). Each of these GLMs produced a correspondingvalue for the explained variance (R^2^), and a winner-take-all approachwas applied such that the model with the highest R^2^was assigned tothat voxel. The receptive field location (X, Y) and size that corresponded tothe best fitting GLM were then linked to each voxel, which was also used tocompute the associated eccentricity and polar angle values. For visualizationpurposes, pRF maps were displayed in cortical surface space and were thresholdedat R > 0.2 (rather than R^2^). Cluster sizes were reported aftercluster extent thresholding to correct for multiple comparisons (seeSection 2.9).

Human rsfMRI data were analyzed using MRICloud (mricloud.org/) to generatewhole-brain rCVR maps using the same default processing pipeline previouslydescribed for rodent rsfMRI data (seeSection2.5for details) (Chan, Lee, et al.,2022;Chan et al., 2021;Liu et al., 2017,^2022^;Lu et al.,2014). Whole-brain rCVR maps in the registered MNI space wereaveraged between subjects for display. T1-weighted images were also segmentedusing*T1 MultiAtlas Segmentation*in MRICloud with defaultsettings to extract various anatomical ROIs (Fig.2B). Group-averaged rCVR values were extracted from the ROIs coveringthe visual cortex (VC), motor cortex (MC), caudate putamen (CPu), hippocampus(HP), and basal forebrain (BF).

### 2.9 Statistical analysis

Statistical analysis was performed using a combination of MATLAB, SPSS, andGraphPad PRISM. In general, results are presented as mean ± standarderror of the mean (S.E.M.) across subjects. Unpaired and paired student’s*t*-tests were applied with a false discovery rate orBonferroni correction for multiple comparisons. For scatter plots, simple linearregression was employed to test significance. Results are considered significantwhen*p*< 0.05. We denote *p< 0.05, **p< 0.01, and***p< 0.005. For mouse ofMRImaps, a Student’s*t*-test was performed to identifyactivated voxels using the threshold*p*< 0.001, falsediscovery rate corrected for multiple comparisons. Human pRF maps are displayedat a threshold of R > 0.2 (square root of variance explained of the bestfitting model), corrected for multiple comparisons using cluster extentthresholding (Woo et al., 2014). Theminimum cluster size was derived empirically using Monte Carlo simulations (1000iterations) withα= 0.05. All values described here are default parametervalues provided in the BrainVoyager software and the ClusterThresh plugin.

## 3 Results and Discussion

### 3.1 NORDIC PCA for rodent ofMRI and rsfMRI

**Fig. 3.**
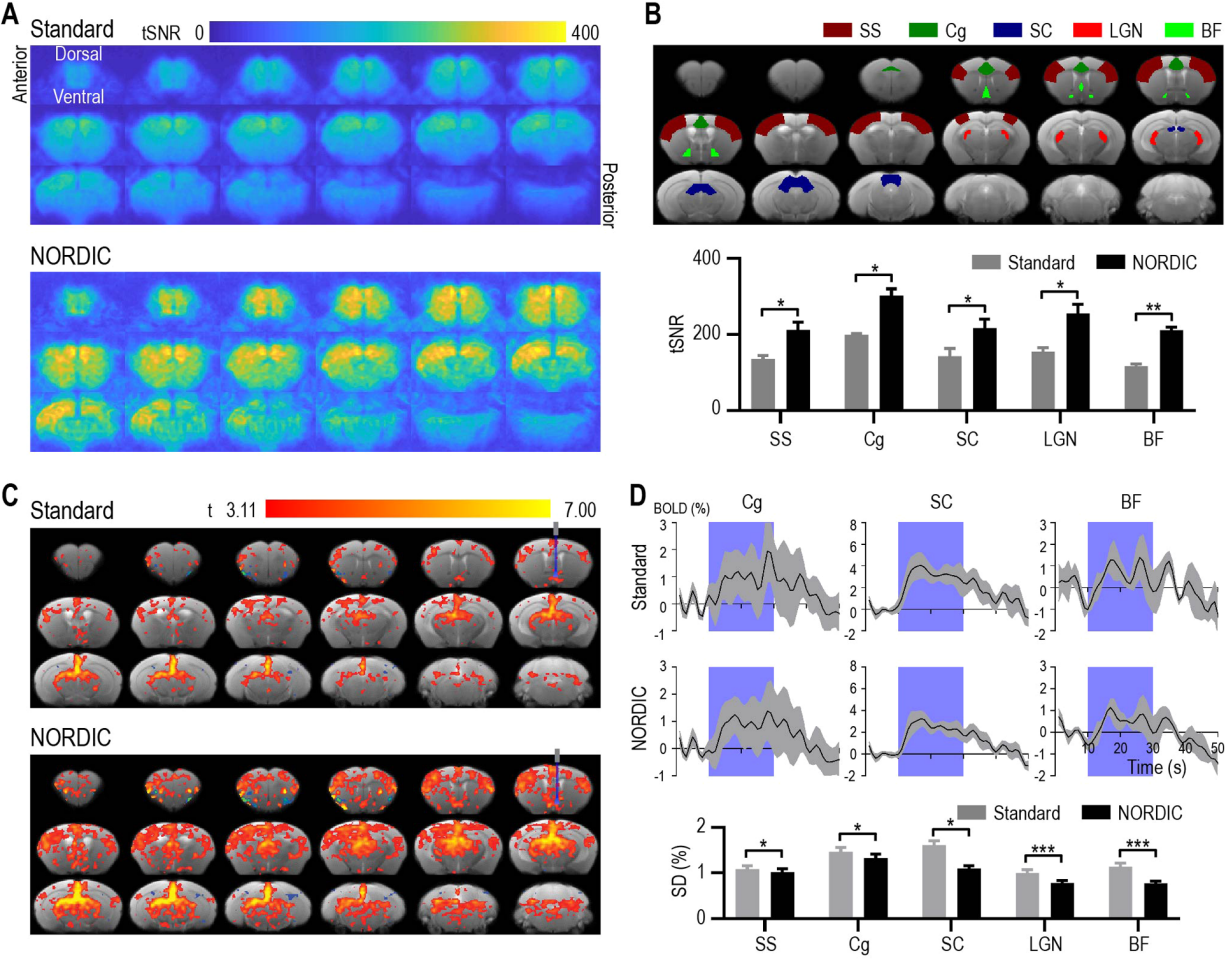
NORDIC PCA increased the temporal signal-to-noise ratio (tSNR) and numberof activated voxels, and decreased signal variation in optogenetic fMRI.(A) tSNR maps of optogenetic fMRI data processed without (Standard) andwith NORDIC PCA. (B) NORDIC PCA increased tSNR in optogenetic fMRIacross different cortical and subcortical brain regions. (C) Group-levelofMRI activation maps illustrating that NORDIC PCA processing induced alarger number of activated voxels compared with Standard processing withthe same statistical*t*-thresholds. Gray and blue barsin the top right image indicate the optical fiber location foroptogenetic stimulation. (D) Group-level ofMRI BOLD timeseries exhibit alower standard deviation (gray areas and bar charts) across differentbrain regions after NORDIC PCA processing. Purple areas indicate theoptogenetic stimulation period. SS: somatosensory cortex; Cg: cingulatecortex; SC: superior colliculus; LGN: lateral geniculate nucleus; BF:basal forebrain. Data in (B) and (D) are presented as mean ±S.E.M. across animals; *p< 0.05,**p< 0.01,***p< 0.005.

ofMRI EPI images showed no apparent morphological change with and without NORDICPCA (Supplementary Fig.1A). Overall, it was apparent that NORDIC-processed ofMRI dataexhibited a dramatic increase in tSNR when compared with Standard processingwithout NORDIC PCA. In particular, when examining key ROIs of the basalforebrain circuit as previously defined, we observed a nearly twofold increasein tSNR (*p*< 0.05, corrected multiple paired*t*-test;Fig. 3A-B).Furthermore, the fMRI GLM maps exhibited more activated voxels when using NORDICPCA compared with Standard processing under the same statistical thresholds(Fig. 3C). In fact, since the basalforebrain is considered the major cholinergic output of the central nervoussystem with projections to many cortical and subcortical regions (Li et al., 2018), we expected widespreadbrain activation similar to the NORDIC PCA activation map. To further confirmthat NORDIC processing did not compromise spatial specificity, we examinedNORDIC and Standard activation maps across various*t*-valuethresholds (SupplementaryFig. 2). In doing so, we found that many areas of activation observedin NORDIC maps at strict statistical thresholds also appeared in Standard mapsas the statistical thresholds were reduced. We also computed brain-wide percentsignal change (PSC) maps for both processing methods (Supplementary Fig. 3).Similar to recent works, Standard and NORDIC processing induced similar PSCvalues, with minor and focal reductions for NORDIC (Dowdle et al., 2023;Faes et al., 2024;Vizioli et al.,2021). When taken together, it is important to note that elevated*t*-values on NORDIC maps did not colocalize with elevatedPSC values. These observations suggest that NORDIC does not notably compromisespatial specificity while increasing the sensitivity of detecting neuralactivation.

The BOLD timeseries also exhibit a lower standard deviation after the applicationof NORDIC PCA in various ROIs (*p*< 0.05, correctedmultiple paired*t*-test;Fig.3D). These results indicated that NORDIC PCA not only successfullydenoised the ofMRI data, but also stabilized the temporal response ofoptogenetically evoked brain activity. To further compare NORDIC PCA withsimilar denoising methods targeting thermal noise, we also computed whole-braintSNR values for the current ofMRI dataset after MPPCA processing (kernel size= [7 7 7] for both approaches). We found that NORDIC PCA and MPPCA bothexhibited a significant improvement over Standard processing, but resulted innearly identical values at the whole-brain and individual ROI level (Supplementary Fig.4A-B).

**Fig. 4.**
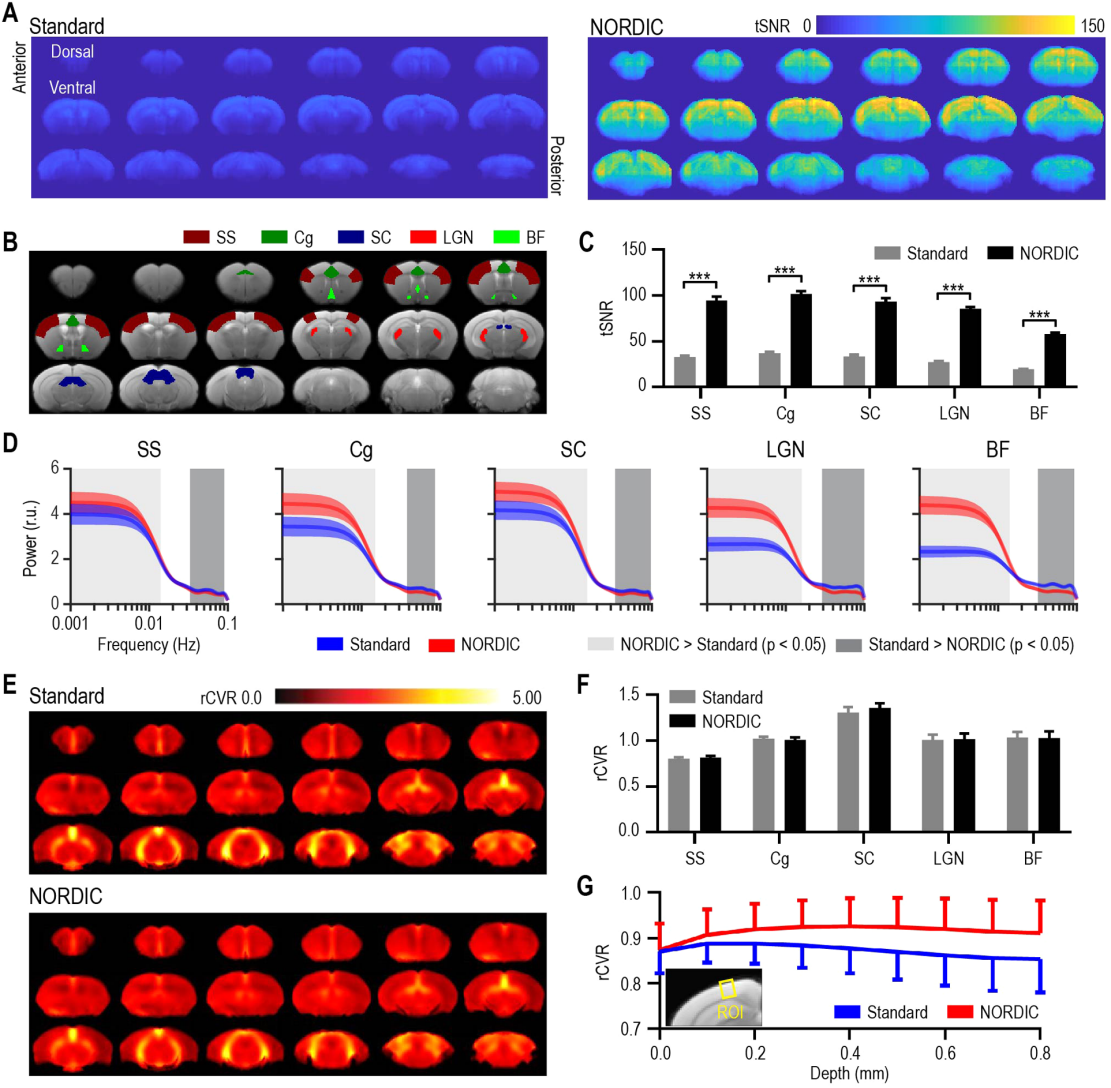
NORDIC PCA increased tSNR and suppressed high-frequency noise in rodentresting-state fMRI, and improved task-free relative cerebrovascularreactivity (rCVR) along cortical layers. (A–C) NORDIC-processedresting-state fMRI data exhibited higher tSNR values across differentcortical and subcortical brain regions when compared with Standardprocessing. (D) When evaluating the group-level power spectra of theBOLD timeseries, all ROIs exhibited increased relative power with NORDICPCA processing (red curve) compared with Standard processing (bluecurve) for frequencies lower than ~0.01 Hz. Similarly, the relativepower for NORDIC PCA processing was lower than Standard processing forall ROIs at frequencies higher than ~0.03 Hz. (E–F) Group-levelrCVR maps showed no apparent morphological or numerical changes withNORDIC processing at the whole-brain or ROI level. (G) NORDIC PCAsignificantly increased rCVR along the cortical depth. SS: somatosensorycortex; Cg: cingulate cortex; SC: superior colliculus; LGN: lateralgeniculate nucleus; BF: basal forebrain. Data in (C), (D), (F), and (G)are presented as mean ± S.E.M. across animals; r.u.: relativeunit. For (C), ***p<0.005, and for (D) the light gray patch indicates NORDIC >Standard,*p*< 0.05, Bonferroni corrected, andthe dark gray patch Standard > NORDIC,*p*< 0.05, Bonferroni corrected.

rsfMRI EPI images also showed no apparent morphological change with and withoutNORDIC PCA (SupplementaryFig. 1B). NORDIC-processed rsfMRI data revealed a higher tSNRcompared with Standard processing without NORDIC PCA by a factor of more thanthree (*p*< 0.001, corrected multiple paired*t*-test;Fig. 4A-C),indicating that NORDIC PCA was successful at denoising the fMRI data andpreserving signal integrity. In fact, such an improvement (>3 times) mayenable experiments in which only low SNR and high-resolution fMRI data areavailable (Vizioli et al., 2021). It isimportant to note that the tSNR of the rsfMRI data here is lower (for NORDIC andStandard cases) than that of the previously described ofMRI data. Thisdifference in tSNR between the ofMRI and rsfMRI datasets is likely due todifferences in (1) the voxel size (0.047 mm^3^vs. 0.02mm^3^), (2) the receiver coils used (surface coil vs. BrukerCryoProbe), (3) the receiver bandwidth (3125 Hz vs. 2500 Hz), and (4) the numberof frequency- and phase-encoding steps (i.e. matrix size, 64 x 64 vs. 80 x 35).Nevertheless, all of the values reported here fall within normal ranges reportedthroughout the literature (Edelman et al.,2021;Grandjean et al., 2020;Jung et al., 2019). Despite thesedifferences, we specifically attempted to optimize the raw tSNR values using arelatively low TE (12 ms) (seeSection 2.4)(Bhavsar et al., 2014), rather thanoptimizing BOLD signal change with a higher TE (Han et al., 2019). We chose to optimize SNR to both ensure that theGaussian assumption of NORDIC PCA holds and to follow recent trends from humanNORDIC work that focus on paradigms such as multiband imaging and laminar fMRIthat also aim to maximize SNR (Dowdle et al.,2023;Knudsen et al.,2023).

When comparing the power spectra in specific brain areas, we found across allROIs that NORDIC PCA induced a significantly higher relative power in lowfrequencies below ~0.01 Hz when compared with Standard processing (Fig. 4D, light gray bars,*p*< 0.05, Bonferroni corrected). Accordingly, we also found in the sameROIs that NORDIC PCA induced a lower relative power in high frequencies greaterthan ~0.03 Hz (Fig. 4D, dark gray bars,*p*< 0.05, Bonferroni corrected). Interestingly, wefound the strongest effects in the BF and LGN, which are much smaller regionsthan the other ROIs included here. Accordingly, these regions are moresusceptible to noise at high spatial resolution, and it is expected that thepreserved spatial precision of NORDIC PCA would impact these regions to greaterextent than larger ROIs. Other resting-state studies investigating similarinfraslow BOLD profiles suggest that a large portion of spontaneous brainactivity can be explained by coactivation patterns (CAPs), which are thought torepresent fluctuating brain-wide states (Gutierrez-Barragan et al., 2019,^2022^). While more general functional connectivity analysis canbenefit from frequency content up to 0.20–0.25 Hz using anestheticprotocols similar to that used in the current work (Cabral et al., 2023;Grandjean et al., 2014), CAPs specifically exhibit a dominantfrequency content below 0.03 Hz. Therefore, the overall shift that we observe inthe content of regional power spectra toward lower frequencies suggests that theapplication of NORDIC PCA may help to better detect CAP profiles and to identifytheir time-varying organizing principles.

The average whole-brain rCVR maps and ROI-specific rCVR values exhibited noapparent changes with and without NORDIC PCA (Fig.4E-F), which support previous studies that report little effect ofdenoising strategies on rCVR values (Cohen etal., 2021;Moia et al., 2021).Nevertheless, Levene’s F-test showed that NORDIC PCA processing(σ²: 0.0116) induced a significantly lower variance in rCVR valuescompared with Standard processing (σ²: 0.0144,*p*< 0.05). The lack of whole-brain differences led us to hypothesize thatthe effect of NORDIC PCA on rCVR may lie at a finer spatial scale than a fullanatomical ROI. As such, we examined the rCVR values as a function of corticaldepth (not layer specific) and found that Standard processing led to a gradualdrop in rCVR values with increasing depth. By contrast, NORDIC PCA preservednearly constant rCVR values across all depths (*p*<0.0001, one-way ANOVA;Fig. 4G). Since rCVRis thought to reflect the conditions of capillaries, as they are the primarysite of oxygen exchange in the brain, we expect a constant rCVR across allcortical depths as the neocortex consists of a uniform capillary bed (Kirst et al., 2020). In this sense, itappears that rCVR values extracted from NORDIC PCA in the cortex better reflectthe underlying physiology (Fig. 4G). Withthis being said, as rCVR was originally developed and validated in humans, weare applying it here to mouse data as vasodilation is thought to beevolutionarily conserved across these two species. While this assumption issufficient to begin exploring rCVR in mice, future studies should extend thisinvestigation by validating rCVR through the direct manipulation ofCO_2_content, perhaps by manually changing either the ventilationrate or the amount of CO_2_being inhaled. Nevertheless, these resultsadd to the growing body of work investigating how rCVR values may be impacted byfMRI signal quality, and suggest that such information examined at a finespatial scale (i.e. across cortical depth) may in fact be affected by NORDIC PCAand higher tSNR.

**Fig. 5.**
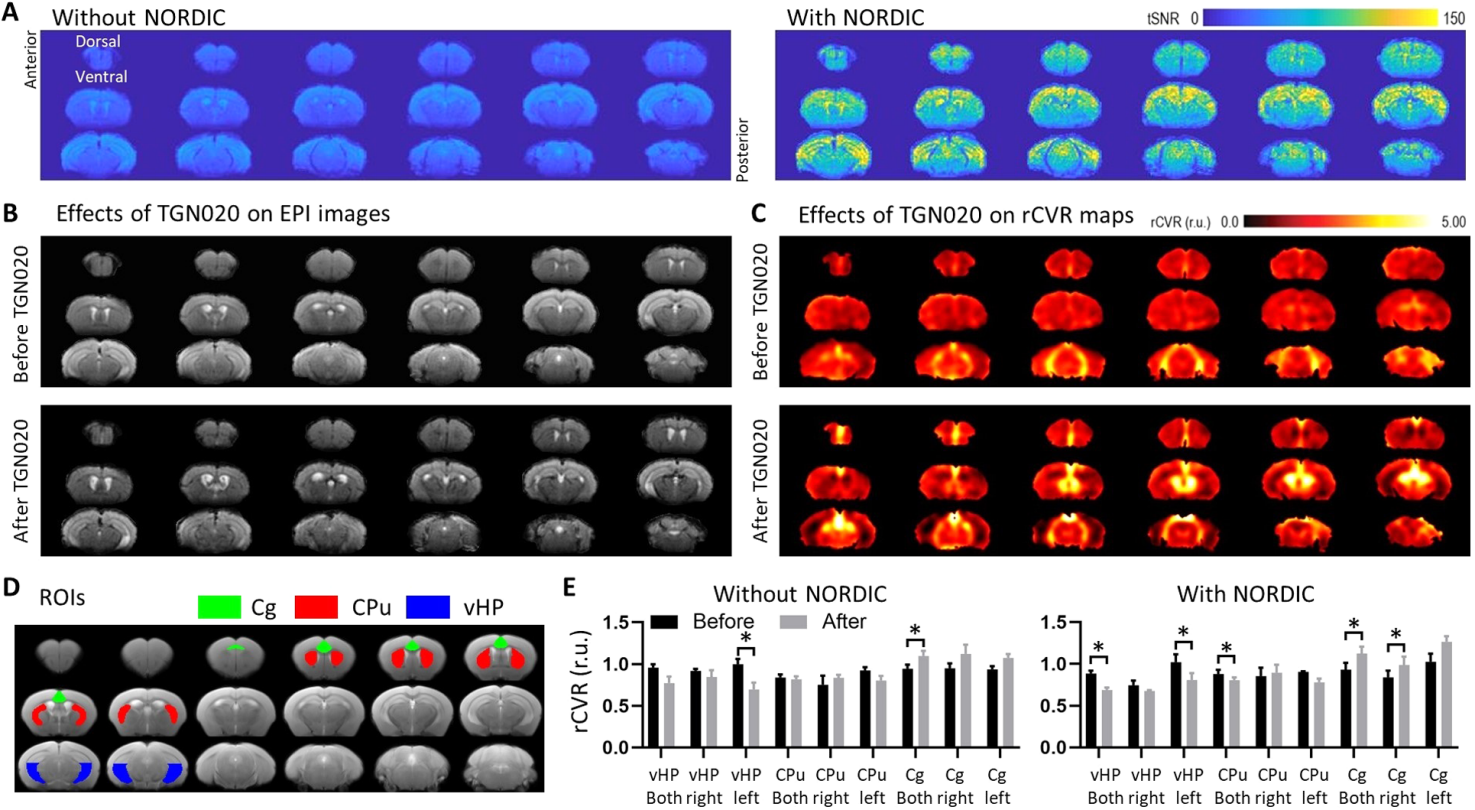
NORDIC PCA improved the detection of TGN020-induced aquaporin-4inhibition on rCVR. (A) NORDIC-processed resting-state fMRI data foraquaporin-4 inhibition experiments exhibited higher tSNR when comparedwith Standard processing. (B) NORDIC-processed resting-state fMRIecho-planar imaging (EPI) of a representative mouse before and afterintrathecal TGN020 infusion showed no major EPI distortion. (C) rCVRmaps of a representative mouse before and after intrathecal TGN020infusion revealed quantitative rCVR changes without apparentmorphological change. (D–E) rCVR significantly decreased in theleft vHP and increased in bilateral Cg after intrathecal TGN020 infusionboth with and without NORDIC PCA. Furthermore, rCVR significantlydecreased in the bilateral vHP and CPu, and increased in the right Cgafter intrathecal TGN020 infusion only after NORDIC PCA. These resultsindicate that NORDIC PCA improved the detection of TGN020-inducedaquaporin-4 inhibition on rCVR. CPu: caudate putamen; Cg: cingulatecortex; vHP: ventral hippocampus. Data in (E) are presented as mean± S.E.M. across animals; r.u.: relative unit;*p< 0.05.

Similar to rsfMRI, the EPI images for the TGN020-induced AQP4 inhibitionexperiments showed no apparent morphological change with and without theapplication of NORDIC PCA (Supplementary Fig. 1C). In addition, TGN020 injection did not induceapparent EPI distortions or morphological rCVR changes (Fig. 5A-B). After intrathecal TGN020 infusion, rCVRsignificantly decreased in the left vHP and increased in the bilateral Cg, bothwith and without NORDIC PCA (*p*< 0.05, paired*t*-test;Fig. 5C-E).Furthermore, rCVR significantly decreased in bilateral vHP and CPu, andincreased in the right Cg after intrathecal TGN020 infusion with NORDIC PCA(*p*< 0.05, paired*t*-test;Fig. 5C-E) but not when analyzed withoutNORDIC PCA (*p*> 0.05). NORDIC is known to reduce signalvariance rather than signal intensity (Vizioliet al., 2021), the latter of which affects rCVR measurements. Whilesignal intensity is not changed by NORDIC (Supplementary Fig. 3),the reduction in variability likely affects the*ΔBOLD/BOLD*term of the rCVR equation, andfacilitates the delineation of changes in rCVR as significant. With this beingsaid, these findings highlight the heterogeneity of AQP4 function across thecerebral vasculature, which may explain the dissimilar vulnerability of variousbrain regions to cerebrovascular diseases.

Moreover, these results were reassuring based on the known mechanism of AQP4 inwhich interaction with Virchow–Robin space water dynamics describes aphysiological model for neurovascular coupling and glymphatic flow (Nakada et al., 2017). Specifically, AQP4mediates water transfer between astrocytes and pericapillary space, and whenTGN020 inhibits this process, water transfer is halted and leads to vasodilationthat can be detected using a cerebral blood flow or even BOLD fMRI contrast(Igarashi et al., 2013;Komaki et al., 2020). As rCVR measures theability of blood vessels to dilate in response to changes in blood flow/volume,we would expect these values to also change in response to TGN020 infusion.Importantly, we observed no difference in the rCVR values in these same ROIseither before or after TGN020 infusion when the data were processed with orwithout NORDIC PCA (Supplementary Fig. 5). These results support the same conclusionfrom the rsfMRI dataset (Fig. 4E-F) in thatNORDIC PCA does not alter rCVR measurements. Nevertheless, when comparing rCVRvalues before and after TGN020 infusion, we found that data processed withNORDIC PCA exhibited more significant differences compared with Standardprocessing (Fig. 5E), suggesting thatNORDIC PCA improves the detection of TGN020-induced AQP4 inhibition on rCVR.

### 3.2 NORDIC PCA for human pRF fMRI and rsfMRI

**Fig. 6.**
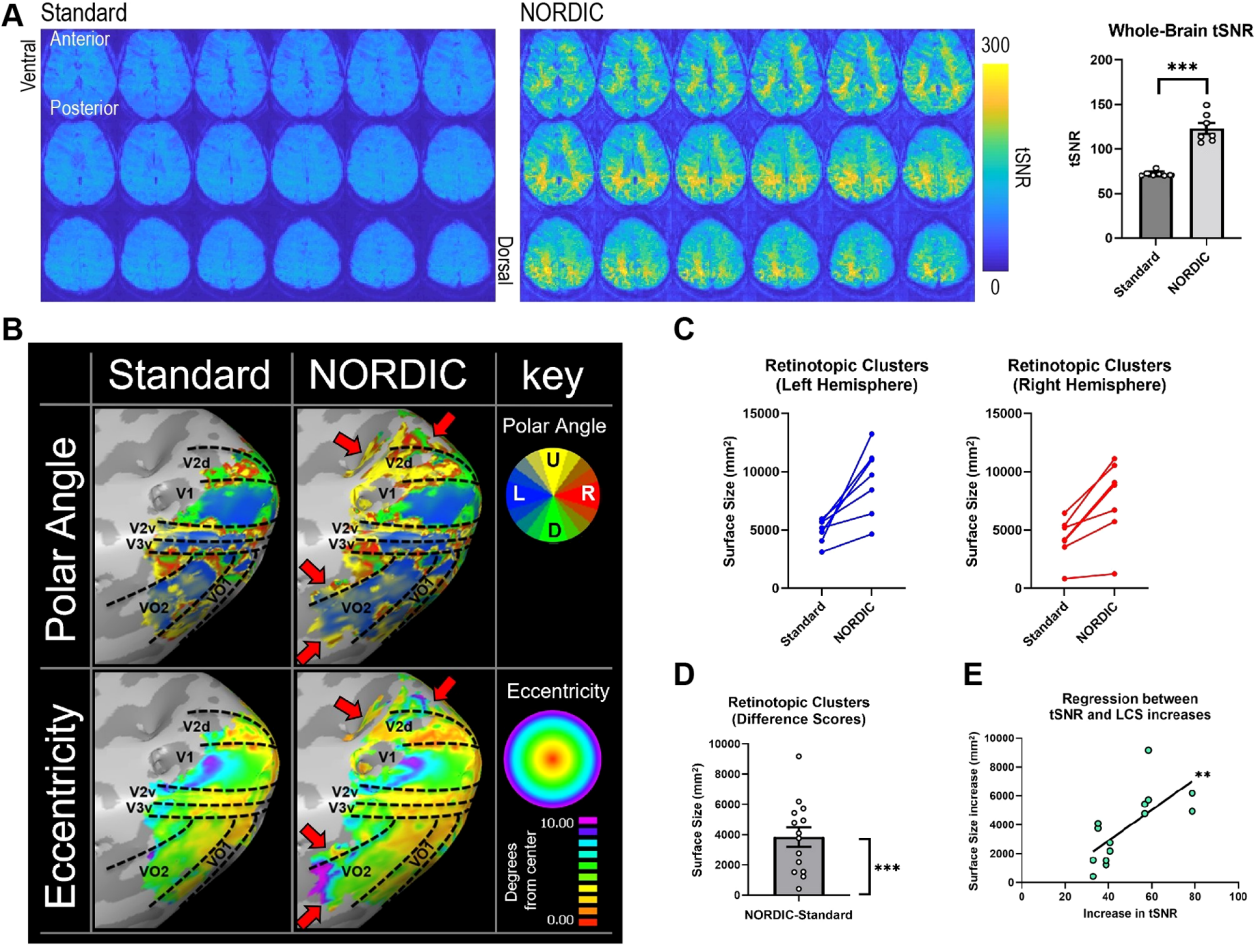
Statistical analyses of task-based fMRI data, examining the effects ofNORDIC PCA on tSNR, retinotopic analysis, largest cluster sizes (LCS),and correlation between tSNR and LCS. (A) Whole-brain tSNR valuessignificantly increased with NORDIC PCA compared with Standardprocessing. (B) Retinotopic cortical organization in a singlesubject’s right hemisphere for polar angle (top row) andeccentricity (bottom row). Cortical delineation was performed withreference to a previous study (Henriksson et al., 2012). At a default threshold of R> 0.2, the active regions that were shared across Standard andNORDIC-processed data exhibited similar retinotopy. However, NORDIC PCAmaps were clearly expanded compared with Standard maps, with moreareas/voxels reaching statistical significance at the same statisticalthreshold (red arrows). U: Up, D: Down, L: Left, R: Right. (C) LCS forStandard and NORDIC-processed data prior to default BrainVoyagerpreprocessing and pRF mapping are shown for the left and righthemispheres. (D) The LCS difference score (NORDIC minus Standard) wassignificantly greater than zero. (E) tSNR was significantly correlatedwith LCS for data processed with NORDIC PCA. Data in (A) and (D) arepresented as mean ± S.E.M. across subjects and clusters,respectively; **p< 0.01,***p < 0.005.

In task-based fMRI using a visual stimulation paradigm for pRF modeling,whole-brain tSNR significantly increased when using NORDIC PCA compared withStandard processing (*t*(6) = 7.784,*p*= 0.000237, d = 3.75), increasing from a mean of 70.33± 1.27 to 119.16 ± 6.84 (Fig.6). Levene’s F-test revealed that this increase was notuniform with a statistically significant increase in tSNR variability followingNORDIC processing (*F*(1,12) = 18.49,*p*= 0.001032) (Fig. 6A).Similar to the rodent ofMRI dataset, here we also compared NORDIC PCA with MPPCA(kernel size = [7 7 7] for both approaches) in terms of whole-brain tSNRvalues. We found that both denoising methods significantly increased global tSNRcompared with Standard processing, but exhibited no significant differencebetween one another (Supplementary Fig. 4C).

fMRI analysis using pRF modeling exhibited similar cortical delineation points inthe cortical surface space for both NORDIC and Standard processing methods.Importantly, there was no apparent deterioration or alteration in the resultingretinotopic mapping for regions that reached statistical significance in bothcases (Fig. 6B). This indicates that NORDICPCA preserves the underlying spatiotemporal precision of BOLD signal changesthat are necessary for effective retinotopic mapping. Furthermore, compared withStandard processing, NORDIC PCA expanded the cortical regions that reachstatistical significance at a consistent statistical threshold (red arrows inFig. 6B). Importantly, voxels whichonly exceeded the statistical significance threshold with NORDIC processingroutinely showed unique preferential responsivity in terms of polar angle and/oreccentricity when compared with neighboring regions that exceeded thestatistical significance threshold in both preprocessing cases. The lack ofspatial blurring across an expanded set of significant and neighboring voxels interms of these characteristics suggests that NORDIC preserves spatialspecificity of pRF mapping while also increasing sensitivity (Fernandes et al., 2023). Another recent studyexamining human tonotopy also found that NORDIC processing preserved tonotopicgradients around the Heshl’s Gyrus (Faeset al., 2024). These comparable effects of NORDIC processing onsensory-based mapping studies further suggest that this approach preservesspatial specificity compared with Standard processing.

The largest cluster sizes (LCS) observed within each hemisphere of the corticalsurface space increased with NORDIC PCA for all subjects compared with Standardprocessing (Fig. 6C). This was furtherconfirmed using the difference scores (NORDICLCS–“Standard” LCS) which revealed that NORDIC PCA induceda significant increase in LCS, with a mean difference of 3833.57 ± 643.2voxels (*t*(13) = 5.960,*p *=0.000047, d = 1.59) (Fig. 6D).Furthermore, the largest LCS observed per hemisphere positively correlated withtSNR increase, as shown using simple linear regression (*F*(1,12)= 11.95,*p *= 0.0048, r^2^=0.4989) (Fig. 6E). This indicates thatlarger increases in tSNR from NORDIC PCA result in larger cluster sizes.

Overall, we show that NORDIC PCA is suitable for pRF modeling by preserving thespatial precision of signals necessary for retinotopic mapping. As a result,more regions with subtle activity changes can be detected with the same or lessstatistical power to improve specificity. NORDIC PCA can be particularlybeneficial for datasets with relatively low tSNR, such as those with a largenumber of volumes, with single subject data, or with data acquired using lowfield strengths. This approach may also help to improve the analysis of dataalready acquired. Taken together, NORDIC PCA exhibits key benefits as acomplementary analysis to improving topographical fMRI mapping.

NORDIC-processed human rsfMRI data exhibited a higher tSNR compared with theStandard processing without NORDIC PCA by more than 50% (*p*< 0.005, corrected multiple paired*t*-test;Supplementary Fig. 6A),indicating that NORDIC PCA successfully denoised the data. The average rCVR mapsand values exhibited no apparent changes with and without NORDIC PCA fordifferent ROIs that include VC, MC, CPu, HP, and BF (Supplementary Fig. 6B).Since lower signal variance is expected in human rCVR with NORDIC PCA, futureclinical studies may benefit from the increased sensitivity of detecting rCVRchanges with NORDIC PCA. Similar studies may also leverage the increase in tSNRto push for the acquisition of rsfMRI data with higher resolution than thecurrent clinical standard.

### 3.3 Technical considerations and future directions

As we included both rodent and human fMRI data in the current work, it is crucialto examine whether and how NORDIC PCA affects data from these two species.Overall, we observed a notable increase in tSNR for all contexts across bothrodent and human datasets. In fact, for fMRI data involving evoked signals, suchas ofMRI in rodents and task-based fMRI in humans, we observed quite a similarpercent improvement (~50%) in tSNR values when using NORDIC PCA compared withStandard processing. For rsfMRI, we observed a similar percent improvement forthe human dataset; however, in the rodent dataset, this improvement was evenmore pronounced (>100%). As NORDIC PCA is intended to denoise withpreserved spatial precision, it is possible that the impact of this approach isstronger on data with higher spatial resolution. While the human datasets wereacquired with similar voxel sizes, the rsfMRI rodent dataset was acquired atover twice the spatial resolution (0.02 mm^3^voxel size) as the ofMRIdataset (0.047 mm^3^voxel size). The higher spatial resolution may,therefore, cause an initially low tSNR that leads to an even larger improvementusing NORDIC PCA. Future studies may benefit from examining the effects of sucha parameter, as well as individual variability and physiological fluctuations,on tSNR improvements with and without NORDIC PCA.

It is also important to note that in the current work, we only applied NORDIC PCAto magnitude images since most fMRI data are stored in magnitude rather thancomplex form. NORDIC PCA was originally introduced for complex images thatexhibit Gaussian noise, since in this context the spectral properties are easilydefined. By contrast, magnitude images exhibit Rician noise; however, recentstudies have re-emphasized that Rician noise can be treated as Gaussian for evenvery low levels of SNR (Fernandes et al.,2023). Nevertheless, it is worth noting that denoising with magnitudedata increases the point-spread function more than when using complex data;however, we must also consider that the previously reported loss of spatialprecision is much less than the blurring induced by other methods (Vizioli et al., 2021). Fortunately, the SNRlevels in the current work are high enough that the Gaussian assumption ofNORDIC PCA still holds for magnitude images. This is notable when comparingNORDIC PCA with MPPCA, which was tested and optimized for magnitude images(Manzano Patron et al., 2024), andalso performs well at high SNR (similar to complex images) (Does et al., 2019). With this being said, magnitudeimages contain spatially correlated noise, which can further accumulate throughthe use of multichannel receiver coils (CryoProbe) and partial Fourieracquisition approaches, and must be accounted for. In this regard, one keyadvantage of NORDIC PCA over MPPCA is that it addresses this confound by mappingthe spatially varying and correlated noise to zero mean and spatially identicalnoise in an individual patch using g-factor normalization (Fernandes et al., 2023;Henriques et al., 2023;Moeller et al., 2021). In fact, this is similar tothe Threshold PCA (TPCA) and General PCA (GPCA) approaches proposed inHenriques et al. (2023)that avoidcorrecting for spatial varying noise by using local noise estimation instead. Insuch work, the application of TPCA was representative of NORDIC PCA and wasshown to be robust across a wide range of conditions that introduce varyingamounts of spatial correlations.

The differences between NORDIC PCA and MPPCA on global tSNR were modest for boththe rodent ofMRI and human pRF fMRI datasets. This relatively small differencein tSNR values is not entirely unexpected as comparisons using*t*-statistics from GLMs observed similarly subtleimprovements with NORDIC PCA compared with MPPCA (Dowdle et al., 2023;Vizioli et al., 2021). Along these lines, we must also acknowledgethe potentially suboptimal NORDIC parameters used for such a comparison in thecurrent work. In the majority of the NORDIC results presented here, we used thedefault 11:1 ratio of spatial to temporal voxels which automatically calculatesan optimal kernel size. However, when directly comparing NORDIC and MPPCA, wechose to use a kernel size of [7 7 7] as these values define the previouslyestablished parameter space for this family of denoising approaches. However,these values were derived using diffusion MRI datasets with around 100 volumes,whereas fMRI datasets often contain many more. As the latter is the case fordatasets included in the current work, it is possible that the fixed kernel sizemay not be ideal for the various fMRI contexts examined here. Nevertheless, toour knowledge, there has yet to be a systematic investigation of theseparameters and we, therefore, utilized default values under the assumption thatfurther optimization would lead to added benefits. Future in-depth workexamining how changing NORDIC parameters affects denoising performance wouldgreatly facilitate optimal use throughout the community.

Finally, tSNR should be considered along with other measures of thermal noisereduction such as spatial precision, which has already been extensively comparedbetween NORDIC PCA and MPPCA (Fernandes et al.,2023;Henriques et al., 2023;Moeller et al., 2021). Therefore,while the expansion of NORDIC PCA to magnitude images here demonstratesadditional value in its flexibility to improve fMRI data stored in a differentand more common format, the datasets consist of relatively small sample sizesand future studies with larger cohorts may more comprehensively clarify the fullpotential of this method. For example, the application of NORDIC PCA tomagnitude data from different preclinical and clinical datasets with low tSNR,such as advanced diffusion MRI modeling (Moelleret al., 2021;Sun et al.,2020), diffusion functional MRI (dfMRI) (Le Bihan et al., 2006), and line-scanning fMRI may help addressfundamental biomedical questions, such as elucidating the intrinsicphysiological sources of the underlying BOLD mechanisms (Toi et al., 2022).

In conclusion, our results demonstrate that NORDIC PCA can improve the detectionof brain activity in both rodent and human fMRI by removing thermal noise, andsubsequently increasing tSNR and reducing signal variance. We provide evidencefor this improvement across a variety of different fMRI contexts that range fromtask-based optogenetic stimulations and topological mapping to task-freecerebrovascular reactivity measurements with and without AQP4 inhibition viaTGN020 infusion. These findings may in the future play an important role inimproving overall fMRI data quality and sensitivity for translational andpreclinical neuroimaging research.

## Supplementary Material

Supplementary Material

## Data Availability

The data and codes that support the findings of this study are available from thecorresponding authors upon reasonable request.
