# Supplementary material for: NOise Reduction with DIstribution Corrected (NORDIC) principal component analysis improves brain activity detection across rodent and human functional MRI contexts
Source: Imaging Neurosci (Camb). 2024 Oct 24;2:imag-2-00325. doi: 10.1162/imag_a_00325 (PMC11506209; doi:10.1162/imag_a_00325)
Supplement: Supplementary Material [file imag_a_00325-supp.pdf]

Supplementary Material:

NOise Reduction with DIstribution Corrected (NORDIC) principal component analysis improves brain activity detection across rodent and human functional MRI contexts

Russell W. Chan[1,2,3,4,5,*], Giles Hamilton-Fletcher[1,3], Bradley J. Edelman[6,7], Muneeb A. Faiq[1,3], Thajunnisa A. Sajitha[1,3], Steen Moeller[8], Kevin C. Chan[1,2,3,9,10,11*]

[1]Department of Ophthalmology, New York University Grossman School of Medicine, New York, NY, USA 10017
[2]Neuroscience Institute, New York University Grossman School of Medicine, New York, NY, USA 10016
[3]Tech4Health Institute, New York University Grossman School of Medicine, New York, NY, USA 10016
[4]E-SENSE Innovation & Technology, Hong Kong, China
[5]Hong Kong Centre for Cerebro-cardiovascular Health Engineering (COCHE), Hong Kong, China
[6]Brain-Wide Circuits for Behavior Research Group, Max Planck Institute of Biological Intelligence, Planegg, Germany, 82152
[7]Emotion Research Department, Max Planck Institute of Psychiatry, Munich, Germany 80804
[8]Center for Magnetic Resonance Research (CMRR), University of Minnesota, Minneapolis, MN, USA 55455
[9]Department of Radiology, New York University Grossman School of Medicine, New York, NY, USA 10017
[10]Department of Biomedical Engineering, Tandon School of Engineering, New York University, New York, NY, USA 10016
[11]Department of Ophthalmology, School of Medicine, University of Pittsburgh, Pittsburgh, PA, USA, 15213

*Co-Correspondence to:
Russell W. Chan, Ph.D. (russ.w.chan@gmail.com)
Kevin C. Chan, Ph.D. (chuenwing.chan@fulbrightmail.org)

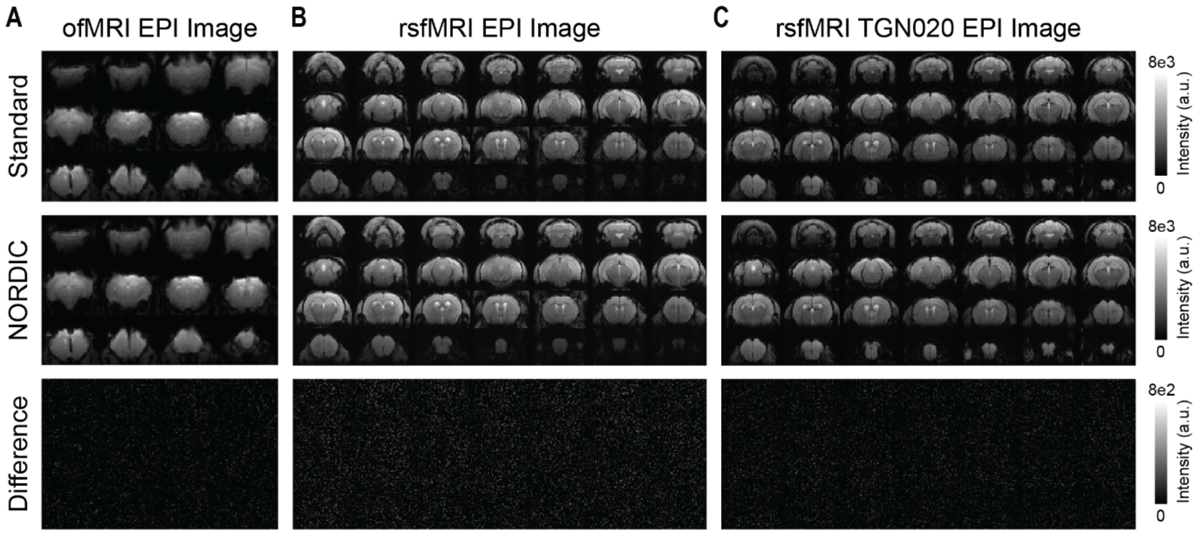

Supplementary Figure 1. NORDIC PCA did not morphologically change EPI images. EPI images exhibited no apparent morphological change with and without NORDIC PCA for **(A)** ofMRI, **(B)** rsfMRI, and **(C)** aquaporin-4 inhibition rsfMRI experiments. The top and middle images showed the same EPI volume before (Standard) and after the application of NORDIC, respectively. The bottom row showed the differences between the Standard and NORDIC volumes, which were minor with no apparent patterns.

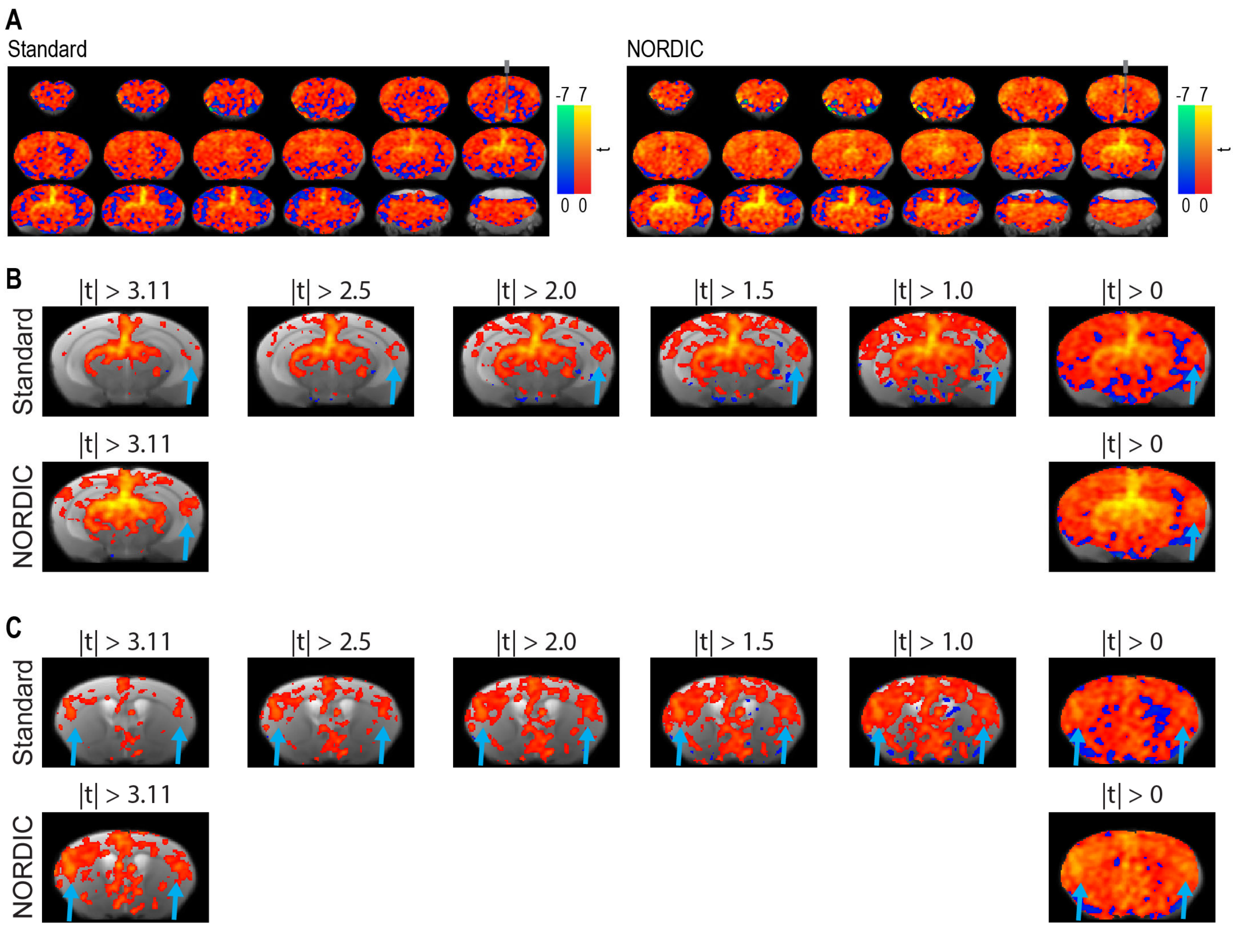

Supplementary Figure 2. Un-thresholded ofMRI activation maps with and without NORDIC processing showed similar patterns at different sensitivity levels. (A) Un-thresholded group-level whole-brain ofMRI activation maps for Standard (left) and NORDIC (right) processing. **(B-C)** Zoomed-in maps of individual slices at Bregma -2.75 mm (B) and Bregma -0.50 mm (C) with progressive t-value thresholds ranging from $|t| > 3.11$ ($p < 0.001$) to $|t| > 0$. Blue arrows highlighted patches of activation that were apparent at the most stringent threshold for data processed with NORDIC, which also progressively appeared for Standard processing as the threshold was reduced.

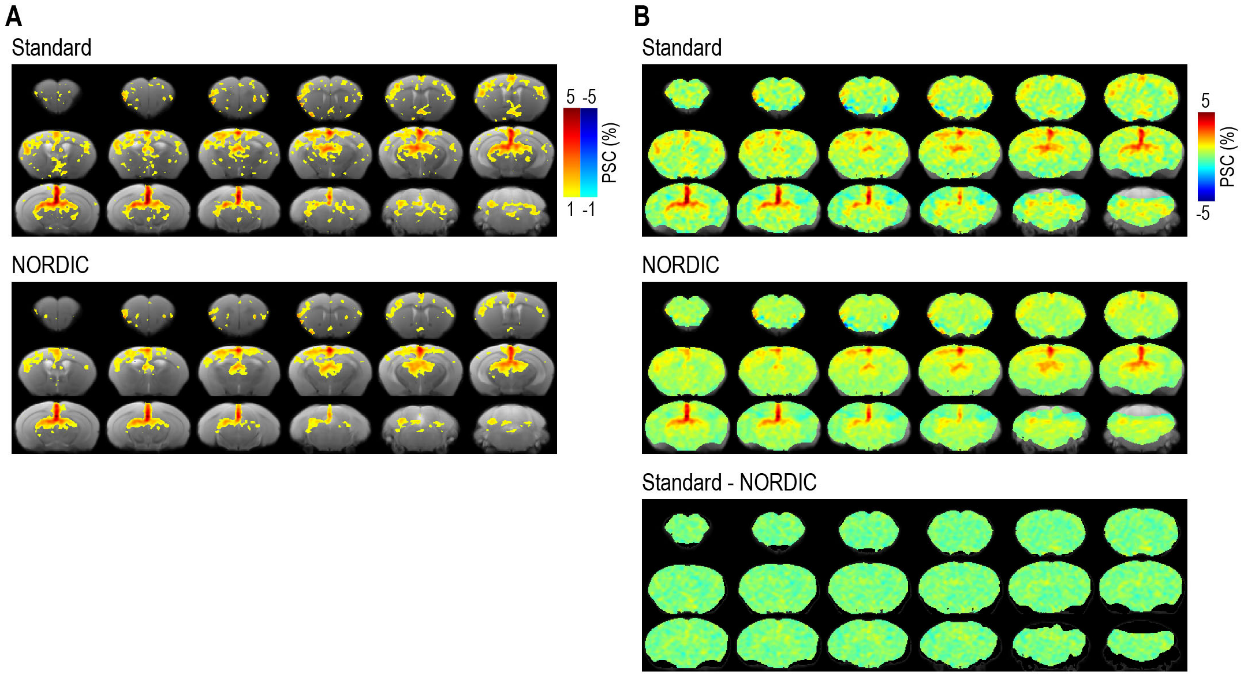

Supplementary Figure 3. Whole-brain percent signal change (PSC) maps were minimally affected by NORDIC PCA. (A) Group-level PSC maps for ofMRI data analyzed using Standard (top) and NORDIC (bottom) processing. Maps were thresholded at a PSC of 1%. **(B)** Un-thresholded PSC maps for Standard (top) and NORDIC (middle) processing. The bottom row showed minor differences between the Standard and NORDIC maps (Standard – NORDIC).

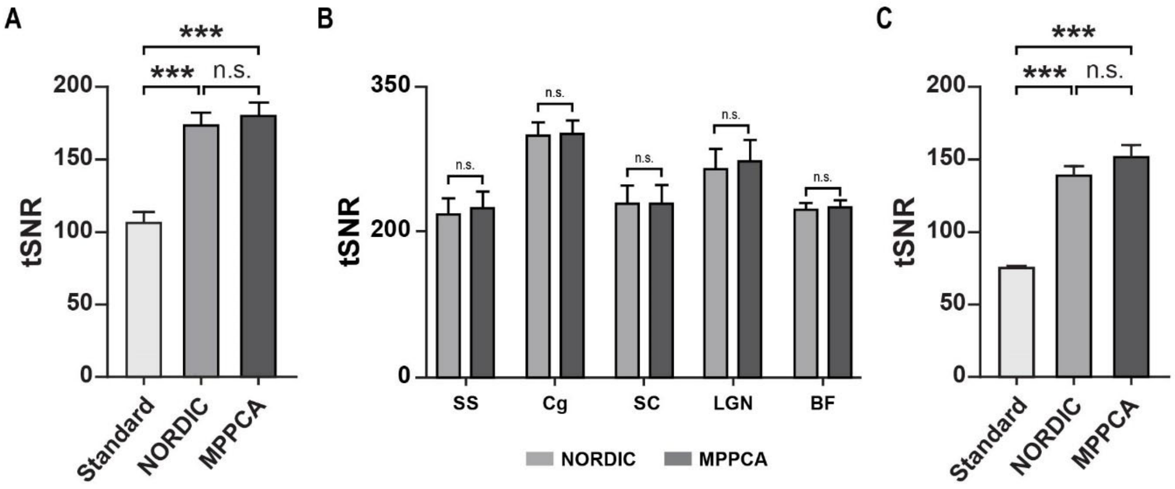

Supplementary Figure 4. NORDIC PCA similarly improves tSNR compared to MPPCA across rodent and human fMRI. (A) When using an identical kernel size optimized for MPPCA (kernel size = [7 7 7]), both NORDIC and MPPCA-processed data exhibited significantly improved whole-brain tSNR compared to Standard processing for the rodent ofMRI dataset (Figure 3). **(B)** A detailed tSNR comparison across different cortical and subcortical brain regions in the ofMRI dataset showed that NORDIC and MPPCA-processed data exhibit similar tSNR values. SS: somatosensory cortex; Cg: cingulate cortex; SC: superior colliculus; LGN: lateral geniculate nucleus; BF: basal forebrain. **(C)** When using an identical kernel size optimized for MPPCA (kernel size = [7 7 7]), both NORDIC and MPPCA-processed data exhibited significantly improved whole-brain tSNR compared to Standard processing for the human pRF dataset (Figure 6). Data are presented as mean ± S.E.M. across subjects. Data were tested with a one-way ANOVA with main effect of method followed by post-hoc pairwise t-tests, *** $p < 0.005$.

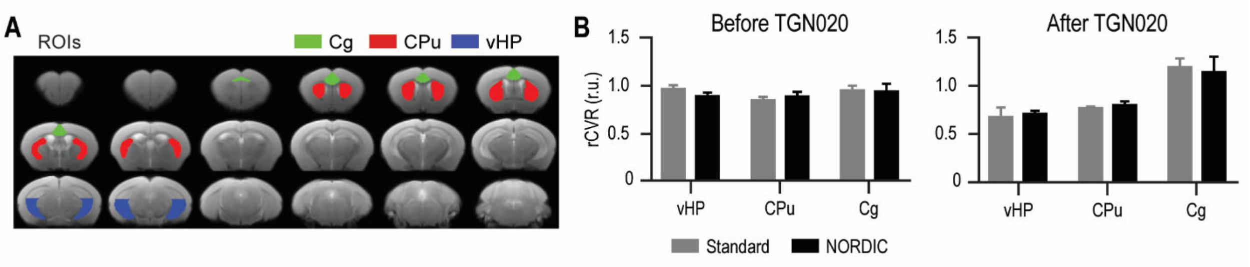

Supplementary Figure 5. NORDIC consistently left rCVR values unchanged compared to Standard preprocessing. Cortical and subcortical ROI-based (A) rCVR values exhibited no apparent numerical change between Standard and NORDIC preprocessed data at timepoints before and after intrathecal TGN020 injection (B). CPu: caudate putamen; Cg: cingulate cortex; vHP: ventral hippocampus. Data in (B) are presented as mean ± S.E.M. across animals; r.u.: relative unit.

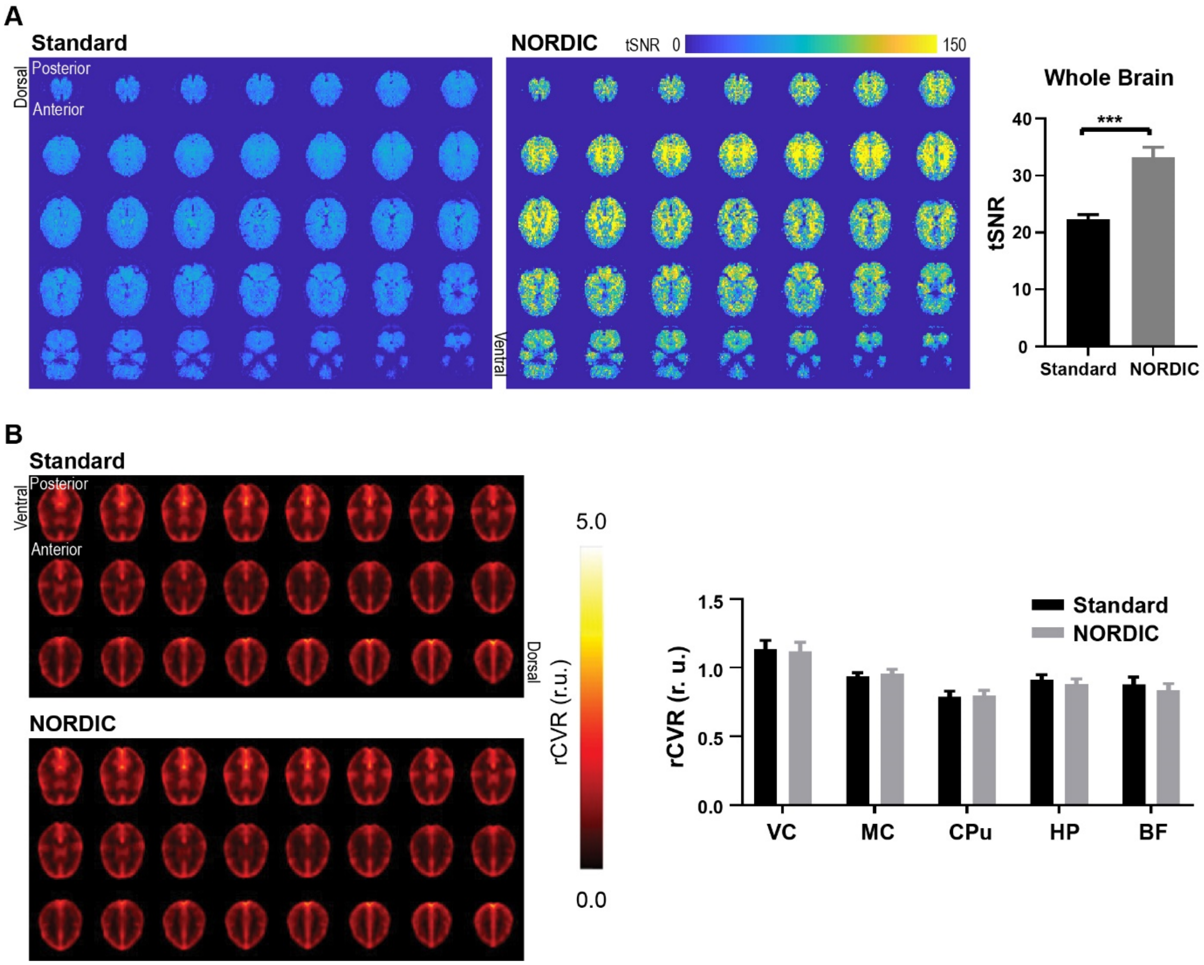

Supplementary Figure 6. NORDIC PCA increased tSNR in human resting-state fMRI with no apparent rCVR changes. (A) Human resting-state fMRI data exhibited a higher tSNR across different cortical and subcortical brain regions when processed with NORDIC PCA compared to Standard processing. **(B)** rCVR maps showed no apparent morphological or numerical change after NORDIC PCA. VC: visual cortex; MC: motor cortex; CPu: caudate putamen; HP: hippocampus; BF: basal forebrain. Data in (A) and (B) are presented as mean ± S.E.M. across subjects; r.u.: relative unit; *** $p < 0.005$.
